# Wavelet gated multiformer for groundwater time series forecasting

**DOI:** 10.1038/s41598-023-39688-0

**Published:** 2023-08-05

**Authors:** Vitor Hugo Serravalle Reis Rodrigues, Paulo Roberto de Melo Barros Junior, Euler Bentes dos Santos Marinho, Jose Luis Lima de Jesus Silva

**Affiliations:** 1 Geological Survey of Brazil - SGB, Avenida Ulysses Guimarães, 2862 Centro Administrativo da Bahia, Salvador, BA 1649-026 Brazil; 2 Petrobras, Petróleo Brasileiro S.A, Av. Repúlica do Chile, No 65 Centros, Rio de Janeiro, 20031-912 Brazil; 3 Research Center in Geophysics and Geosciences, Federal University of Bahia, Rua Barão de Jeremoabo, Ondina, Salvador, BA 40210-630 Brazil; 4 Division of Artificial Intelligence and Integrated Computer Systems, Department of Computer and Information Science, Linköping University, SE-581 83 Linköping, Sweden

**Keywords:** Hydrology, Engineering, Mathematics and computing

## Abstract

Developing accurate models for groundwater control is paramount for planning and managing life-sustaining resources (water) from aquifer reservoirs. Significant progress has been made toward designing and employing deep-forecasting models to tackle the challenge of multivariate time-series forecasting. However, most models were initially taught only to optimize natural language processing and computer vision tasks. We propose the Wavelet Gated Multiformer, which combines the strength of a vanilla Transformer with the Wavelet Crossformer that employs inner wavelet cross-correlation blocks. The self-attention mechanism (Transformer) computes the relationship between inner time-series points, while the cross-correlation finds trending periodicity patterns. The multi-headed encoder is channeled through a mixing gate (linear combination) of sub-encoders (Transformer and Wavelet Crossformer) that output trending signatures to the decoder. This process improved the model’s predictive capabilities, reducing Mean Absolute Error by 31.26 % compared to the second-best performing transformer-like models evaluated. We have also used the Multifractal Detrended Cross-Correlation Heatmaps (MF-DCCHM) to extract cyclical trends from pairs of stations across multifractal regimes by denoising the pair of signals with Daubechies wavelets. Our dataset was obtained from a network of eight wells for groundwater monitoring in Brazilian aquifers, six rainfall stations, eleven river flow stations, and three weather stations with atmospheric pressure, temperature, and humidity sensors.

## Introduction

Groundwater resources^1^ are among the most critical life-sustaining^2^ assets for communities worldwide. Aquifer reservoirs play a crucial role in irrigated agriculture^3^, water supply^4,5^, and industrial development^6^. The groundwater level measurements are vital for water management systems^7,8^ since they indicate availability, accessibility, and possible disruptions^9,10^. Therefore, an accurate forecast of groundwater levels can also provide policymakers with insights for planning strategies and management of water resources that secure sustainable development in different regions^11,12^. These systems are usually integrated across specific areas through wells connected to the main reservoir. However, due to the complexity and nonlinearity in nature, such as weather fluctuations, groundwater recharge and discharge rate of rivers, various topography, human activities such as aquifer reservoir operations, and changes in atmospheric pressure, precipitation, temperature, and distinct hydrogeological conditions and their interactions can profoundly affect the predictions of groundwater levels^13,14^.

Numerous approaches have been proposed for modeling, simulating, and predicting groundwater levels using conceptual models^15^, finite difference^16^, and finite element^17,18^ approaches. Although classical models can be reliable for predictions, large volumes of data are necessary. Furthermore, aquifers have different properties, such as various boundary conditions underlying geological structures, porous media diffusion rates, and topography affecting reservoirs. Physical-based models can track water conditioning to forecast spatiotemporal distributions^19,20^. However, the complexity and computational costs are exceptionally high since the solution of partial differential equations can take several days. Therefore, designing machine learning models to simulate groundwater levels that capture non-linear dynamics of reservoirs by identifying intrinsic patterns in the time-series data without underlying physical processes is paramount for water management systems^21–24^. Physics-informed neural networks have also been used to simulate the physical process governing aquifers^25–27^. Furthermore, advances have been made in deep-learning-based methods for groundwater prediction^28,29^, genetic algorithms^30,31^, support vector machine (SVM)^32–34^, convolutional (CNN) and temporal convolutional network^35,36^, recurrent neural network, gated recurrent unit (GRU) and long short-term memory (LSTM)^37–39^, and graph neural networks based on Wavenets^40,41^ to include spatiotemporal patterns for groundwater forecasting.

Recent progress has been made toward developing deep-forecasting models to tackle the challenge of multivariate time series forecasting^42^. Most of these models initially taught to only optimize tasks in natural language processing are being efficiently adapted for applications in multiple fields^43,44^. The Transformers^45^, architectures based on the self-attention mechanisms, have shown remarkable improvement in quality and performance for various tasks in machine translation and computer vision^46–48^. These models can capture long-range dependencies, interactions, and relationships in sequential data, an intrinsic characteristic of time series. Since transformers for time series is an emerging subject^49^, many variants have been proposed for deep-forecasting^50^, anomaly detection^51,52^, classification^53^, seasonality trends^54^, and data augmentation^55^. The most recent Transform-based models include the Autoformer^56^, which explores the concept of autocorrelation to rewrite the self-attention block. These unique autocorrelation blocks increase robustness and provide faster and more accurate results than the original Transformer. Additionally, the Informer^57^ replaces self-attention with the *Probspace* self-attention mechanism to handle the challenges of quadratic time complexity and memory usage in the vanilla Transformer. Furthermore, since most time series have a sparse representation in Fourier transform, FEDformer^58^ introduces the Frequency Enhanced Block and Frequency Enhanced Attention to expand the original model and achieve an even higher performance in some applications.

This work proposes the Wavelet Gated Multiformer for groundwater time-series forecasting. Our method combines the strength of vanilla Transformer^45^ concepts behind the Autoformer^56^. It also introduces wavelet autocorrelation blocks in the encoder and decoder for denoising the signals. Furthermore, the self-attention mechanism is responsible for computing the relationship between points inside the time series. At the same time, the autocorrelation finds periodicity patterns (trends) inside the time series, and these mechanisms are mixed through a gate (linear combination) into a single encoder to improve pattern recognition. A multi-headed encoder with gate mixing sub-encoders (Transformer and Wavelet Crossformer) can give the decoder a more concise signature of trending signals and improve the model’s predictive capabilities. Multifractal cross-correlation analysis has been used successfully in a range of studies involving time series pattern investigations, including economic trends^59,60^ and climate^61^. This work also includes the multifractal analysis of cyclical patterns across multifractal regimes between pairs of time-series (stations) through the Multifractal Detrended Cross-Correlation Heatmaps (MF-DCCHM)^62^ with Daubechies 4 Wavelets for high-frequency filtering.

**Figure 1:**
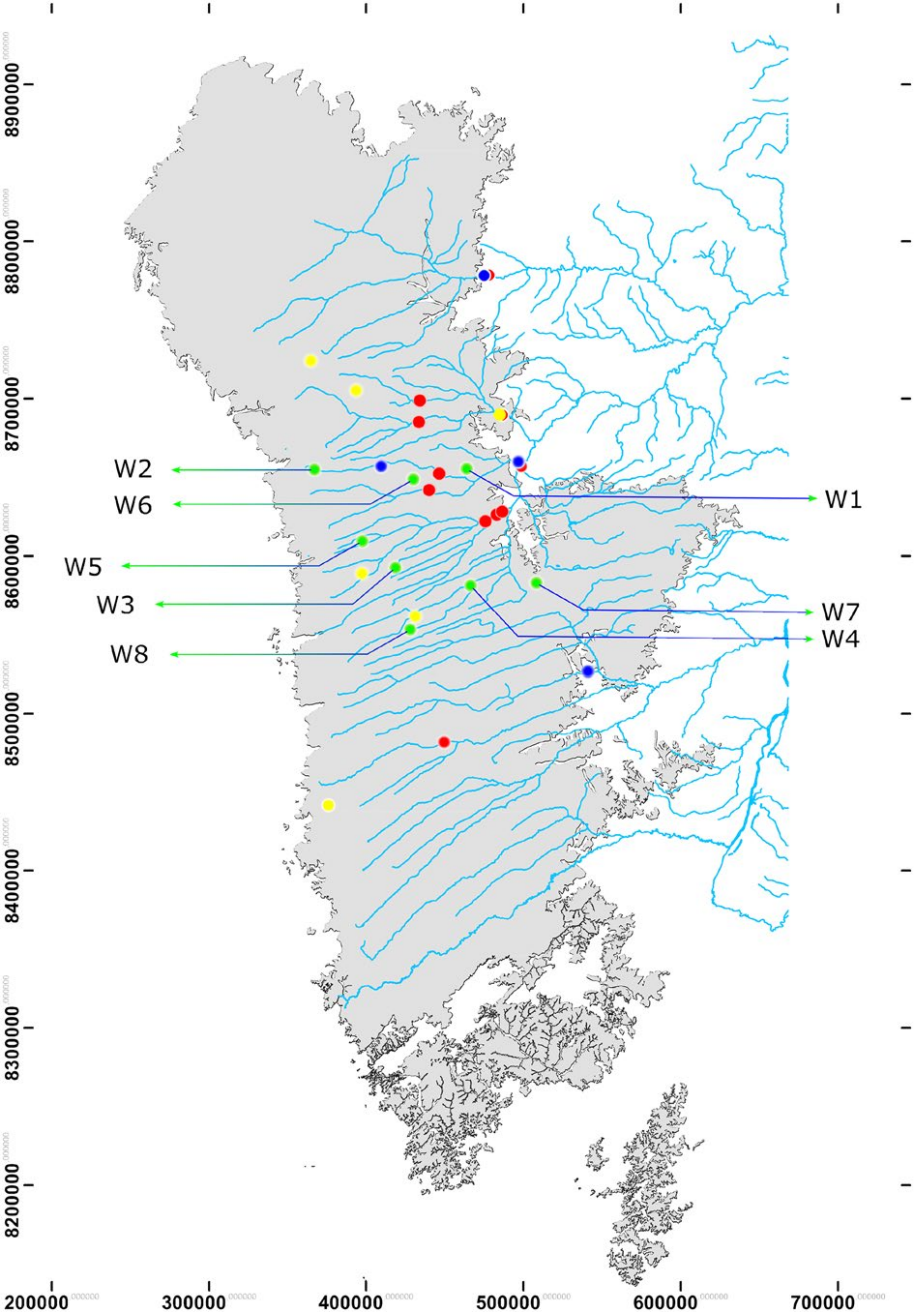
Map diagram with the stations and wells represented in green (W1–W8), rivers stations in red, rainfall stations in yellow and weather stations (atmospheric pressure, humidity, and temperature) in blue. See Table S1 (Supplementary material) for information on latitude and longitude. The map diagram was generated with ArcGIS 10.8^76^ and post-processed with Inkscape^68^ (Open Source Software licensed under the GPL). The dataset is publicly available and can be obtained from the Geoscience system of the Geological Survey of Brazil (GeoSGB)^77^. The license is Creative Commons Attribution NonCommercial 4.0 International.

The Geological Survey of Brazil (SGB) has developed a network of wells for groundwater monitoring in aquifers all over Brazil, also known as the Integrated Groundwater Monitoring Network, or RIMAS. The Urucuia Aquifer System (UAS), located in the west of Bahia state, has over 60 wells for groundwater monitoring and a consistent booming in the agricultural economy over the last decades in its region. The economic boom came with a subsequent increase in demand for water supply. Furthermore, the groundwater from UAS has also been crucial for maintaining the flow of essential affluents of the São Francisco River, the most vital river in the northeast of Brazil. Hence, continued monitoring of groundwater levels in the Urucuia Aquifer is essential. In this work, we have investigated eight wells obtained from a publicly available dataset at RIMAS^63^, six rainfall stations, and eleven river flow stations from the National Hydrometeorological Network (RHN) provided by the Brazilian National Water Agency^64^, and datasets from National Institute of Meteorology (INMET)^65^ with three weather stations including atmospheric pressure, temperature, and humidity sensors (UTM location and alias used for sensors in Tables S1–S4 in Supplementary Materials), as shown in Fig. 1. The data collected has daily sampling and ranges from 1 January 2016 to 31 December 2019. For the station data, we perform aggregation and normalization using an exponential distance factor to reduce the total volume of input data while considering the relative position information of the stations concerning each well. The following section will discuss the main results and their trends.

## Results

### MF-DCCHM for groundwater level pattern recognition

**Figure 2:**
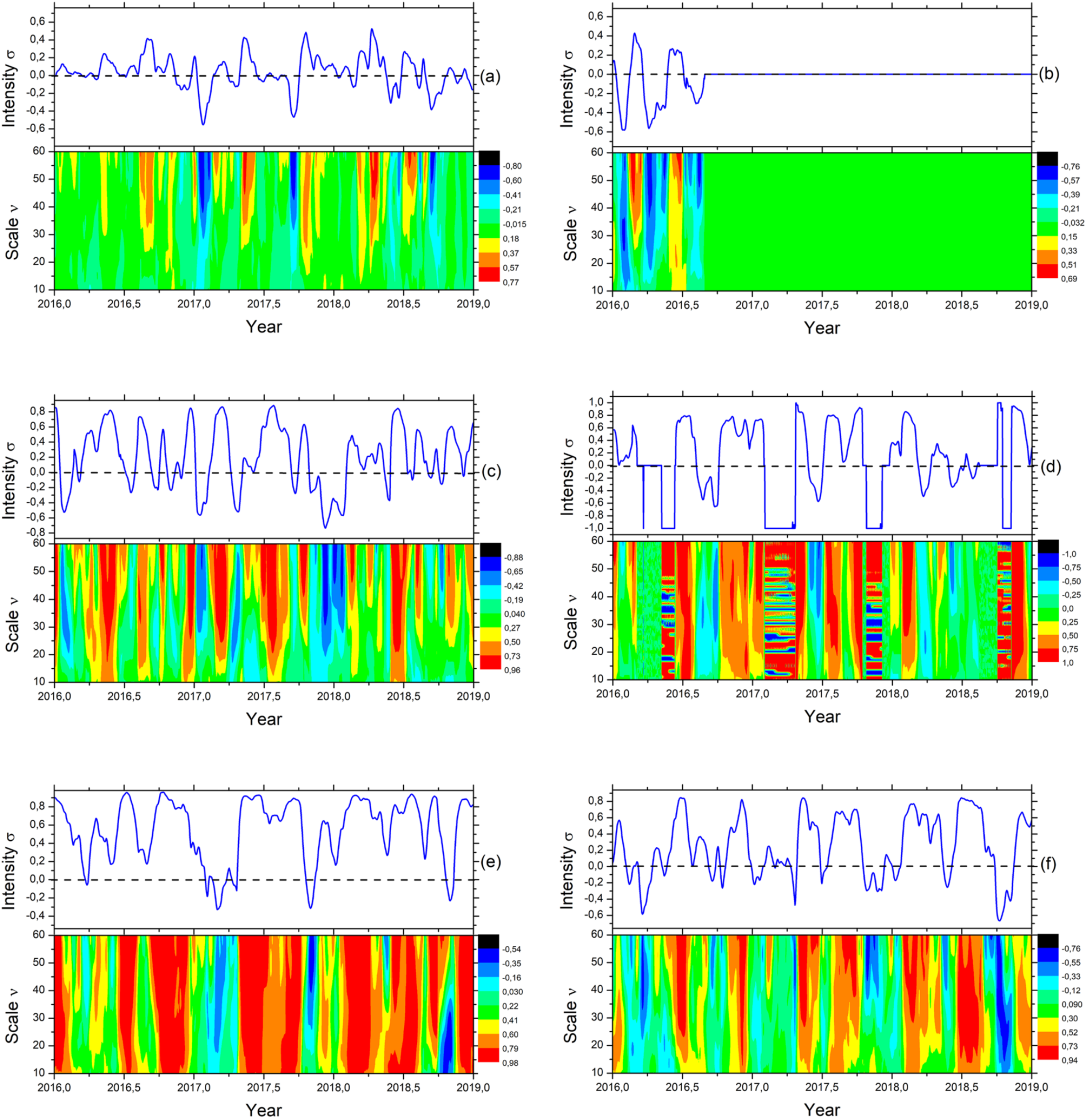
Multifractal Detrended Cross-correlation Heatmaps between the attributes: (**a**) W1 and W8, (**b**) W1 and W6, (**c**) RI2 and RI10, (**d**) R1 and R4, (**e**) T1 and H1 (**f**) T1 and H2.

We have employed the Multifractal detrended cross-correlation heatmaps (details in the “Methods” section), a technique based on the detrended cross-correlation coefficients that can map the relationships between pairs of fluctuations across different multifractal regimes. We have used sliding boxes with sizes of up to 5$\%$ of the entire series for local analysis. After the main computations, the images are generated from tensors and plotted using Python^66^ and matplotlib package^67^. The plots are finally post-processed with Inkscape^68^ to generate the diagrams and heatmaps. These heatmaps can uncover non-explicit cyclic patterns between signals obtained from a combination of sensors under specific constraints. Figure 2 represents the Multifractal Detrended Cross-correlation heatmaps with an average over the intensity of the cross-correlation coefficients for each day on top of the maps. The average can provide a peculiar view of positive and negative trends over multiple regimes considering all possible windows and sliding boxes. The importance of the previous assumption relies on the possibility of uncovering and inferring cyclical patterns across multiple regimes for the time series of groundwater level measured from well sensors as compared to other regional well data, local pressure, humidity data, river levels, or weather attributes such as the local rainfall acquired through sensors from different stations. Our dataset is composed of daily time series from multiple sensors ranging from 1 January 2016 to 2019: (i) eight data series (W1–W8) representing the groundwater levels from the sensor inside the well logs, (ii) six data series to measure the rainfall (R1–R6) from weather stations, (iii) eleven data series (RI1–R11) measuring the river levels, (iv) three data sets representing the atmospheric pressure (P1–P3) in different locations, (v) three data series for the local temperature (T1–T3), and (vi) three data series for the local humidity (H1–H3).

Figure 2a represents the cross-correlation between two wells, W1 and W8. The x-axis represents the temporal variation (daily time series). In contrast, the vertical axis symbolizes the variation of the scale $\nu$ considering the interval $10 \le \nu \le 60$ with a fixed-sized sliding box. The colors correspond to the variation in the intensity of $\sigma$, representing the cross-correlation coefficients between wells W1 and W8. The computed detrended cross-correlation coefficients are in the interval of $-1 \le \sigma \le 1$, as shown in the sidebar of each heatmap. On top, the vertical axis describes the average $\sigma$ (cross-correlation coefficients) for the entire map scope. This chart is crucial since we can focus on identifying cyclical trends due to oscillations and uncover the trend for the entire period (averaged persistence or anti-persistence). These time series will have strong or weak detrended cross-correlated characteristics depending on the color and uniformity, given a vertical range for all scales $\nu$.

The MF-DCCHM for the pair of wells W1 and W8 shown in Fig. 2a displays periodicity for $\sigma >0$ and $\nu > 25$, which represents approximately 6 months by taking $P_{cp}$ as a starting point $\approx$(2016.7;25), where $P_{cp}$ denotes the values with positive cross-correlation coefficients. Additionally, we have identified a cyclical pattern for $\sigma < 0$ and $\nu > 30$, representing approximately 8 months, where the reference value is $P_{cn} \approx$(2017.1;30) for the negative cross-correlation coefficients. On top of the heatmap, the averaged detrended cross-correlation coefficients (vertically) show an exact cyclical pattern due to the oscillations for the entire period. Therefore, the groundwater level in these two wells (W1 and W8) follows a similar trend of fluctuations for the period with positive coefficients. In contrast, its fluctuations have an inverse relationship for negative coefficients. Furthermore, we have found residual cross-correlation coefficients exhibiting weak correlations, hovering around $\sigma \approx 0$.

We have also obtained similar results for all combinations of pairs considered (eight wells) distributed in the region. Our results indicate that the closer the wells are to each other, the greater the probability of these wells following the same fluid volume fluctuations during specific periods. Figure 2b shows the MF-DCCHM for wells W1 and W6, which have a similar pattern as compared to the pair of wells shown in Fig. 2a (W1 and W8) in the time interval between January and August of 2016. However, we have detected anomalies for the entire period of August 2016. Figure 2b shows a uniform green color from 2016 to the end of 2019, with coefficients $\sigma \approx 0$, for any $P \approx$(t > 2016.7; $\nu$), where *t* is the temporal variable. After conducting a detailed analysis of the conditions regarding all the wells, the anomaly in well W6 suggests a massive influx of water due to regular groundwater pumping from a nearby well (around 800 m distant, according to observations in the field).

Figure 2c shows the MF-DCCHM for river two (RI2) and river ten (RI10). Our results indicate direct and indirect proportionality trends related to the fluid volume in different sensors across the rivers for different periods. By analyzing the MF-DCCHM of eleven data series (sensors), we have discovered a standard direct proportionality signature (positive $\sigma$) between the fluctuations in the rivers’ fluid level every 3 months, a characteristic of the region. We have also observed a periodic abnormal pattern in the MF-DCCHM associated with rainfall data series. Figure 2d show the MF-DCCHM for stations R1 and R4, with an atypical pattern between February and March. These periodic intervals coincided with the four bands shown on the map, indicating the non-occurrence of rain in the period.

We have also examined the MF-DCCHM of nine data series from pressure, temperature, and humidity collected in three locations. We have constructed the maps for all pairs of signals. Figure 2e represents the cross-correlation mapping between temperature and humidity series from stations T1 and H1. The map contains cyclical patterns composed of bands in red where $\sigma > 0.8$ for the entire length of the series $10 \le \nu \le 60$, showing a substantial proportionality across all regimes. We have also found the same behavior when comparing temperature at T1, humidity at H1, and rain at R1 with humidity at H1. These high cross-correlated cyclical patterns indicate a signature that the air can saturate under high relative humidity. At a particular temperature, the air is unable to hold water content leading to the formation of clouds and precipitation. The temperature where the air gets saturated (unable to hold moisture) is also known as the dew point. However, we have noticed a weakening of the detrended cross-correlation coefficients affecting the direct proportionality when considering different locations, which can be characterized as a regional effect. Figure 2f shows the MF-DCCHM for temperature and humidity at stations T1 and H1. The average period for direct and inverse proportionality events is approximately 6 months, with attenuation for different distances.

**Figure 3:**
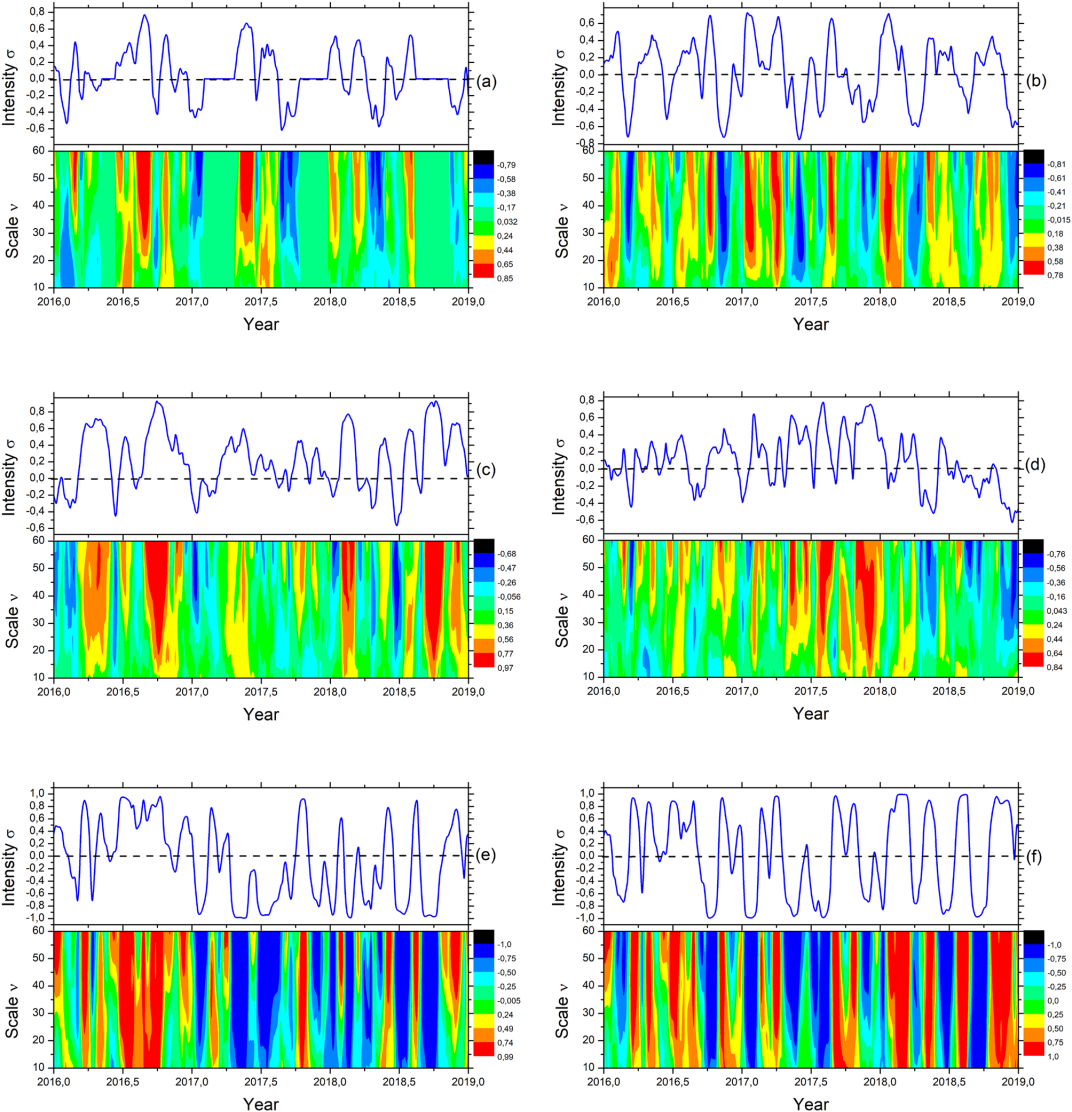
Multifractal Detrended Cross-correlation Heatmaps between the attributes: (**a**) W1 and R4, (**b**) W1 and RI10, (**c**) P1 and T1, (**d**) W1 (Simple Filter) and RI, (**e**) W1 (WaveLet Filter 0.35) and RI and (**f**) W1 (WaveLet Filter 0.70) and RI.

Figure 3a shows the cross-correlations for well W1 and rainfall R4 data series. In addition to the periodicity reported for $\sigma < 0$ and $\sigma > 0$, the maps can show bands composed of the absence of rain with patterns of $0.5\, cycle/year$ considering $\sigma \approx 0$ and $10 \le \nu \le 60$. Figure 3b represents the MF-DCCHM for the pair well W1 and river RI10. This result shows an intrinsic relationship in the cross-correlation between the river and well levels. The closer the well is to the river, the greater the cross-correction between them since interconnection through regional channels can provide different delays of influx and outflux of water, raising groundwater levels at different rates. However, factors such as rainfall intensity in the region considerably impact the affinity between the measurement levels from wells and river stations.

Figure 3d–f were obtained from the pair of variables well and river, including a low-pass filter with Daubechies 4 wavelets. In Fig. 3e, f, we have used a filtering threshold of 0.35 for Fig. 2e and 0.70, respectively. After removing white noise, we can observe an increase in explicit uniform bands across the maps with stronger positive and negative cross-correlation periodicities. Therefore, by reducing high-frequency fluctuations, our signal carries seasonal trends characteristics of low frequencies, which can be relevant for determining the cycles. The filtering helps uncover significative trends extending across multiple regimes since extracting white noise generates more uniform distributions along the vertical axes. It is crucial to highlight the influence of high-frequency fluctuations for pattern recognition and the sensitivity of the MF-DCCHM for capturing seasonal trends after filtering the noise.

Figures S8 and S9 (Supplementary Material) show the $DFA_0$ and $DFA_1$ analyses for all the series in Figs. 2 and 3. The vertical axis represents the fluctuation, the horizontal axis represents the window size, and the vertical bar on the side shows the symbol and color associated with each attribute. In Figs. 2 and 3, the MF-DCCHM captured signatures of two regimes. We have also obtained similar curves for the $DCCA_0$ and $DCCA_1$ analyses. These regimes can be described by the multiscale exponents shown in Tables S5–S13. These results show the autocorrelation and cross-correlation exponents with indications of negatively correlated ($0.0< \alpha < 0.5$), close to uncorrelated ($\alpha \approx 0.5$), and positively correlated ($0.5< \alpha < 1.0$) patterns. Tables 1 and 2 also show an exponent of $\alpha > 1.0$ for well W8 in the second regime represented by region II. For the $DFA_1$, we have found river RI35 in region I and river RI70 in both regions I and II. We have also obtained a multifractal exponent of $\alpha > 1.0$. The occurrence of $\alpha > 1.0$^69^ can also be associated with levels of high-frequency noise in the linear trend. Therefore, we have employed the Daubechies 4 low-pass filter and managed to amplify critical cyclical signatures, denoise the fluctuations, and, in the case of well W8, reduce the multifractal exponents for less than one. That is a highly significant result since we have shown that the MF-DCCHM is very sensitive to high-frequency noise, and denoising with specific thresholds can assist in uncovering low-frequency trends.

**Table 1:** Cross-correlation exponents considering wells W1 and W8.

Method/time-series	Region I	Region II
${DFA}_1$/Well 1 (W1)	$0.115 \pm 0.005$	$0.54 \pm 0.02$
${DFA}_1$/Well 8 (W8)	$0.35 \pm 0.02$	$1.26 \pm 0.02$
${DFA}_0$/Well 1 (W1)	$0.50 \pm 0.02$	$0.976 \pm 0.003$
${DFA}_0$/Well 8 (W8)	$0.944 \pm 0.003$	$0.810 \pm 0.009$
${DCCA}_1$/W1 and W8	$0.181 \pm 0.006$	$0.90 \pm 0.01$
${DCCA}_0$/ W1 and W8	$0.72 \pm 0.01$	$0.042 \pm 0.002$
${DFA}_1$/Well 1 (W1) Wavelet Filter 0.35	$0.115 \pm 0.005$	$0.54 \pm 0.02$
${DFA}_1$/River	$1.46 \pm 0.02$	$0.76 \pm 0.01$
${DFA}_0$/Well 1 (W1) Wavelet Filter 0.35	$0.50 \pm 0.02$	$0.976 \pm 0.003$
${DFA}_0$/River	$0.74 \pm 0.02$	$0.47 \pm 0.01$
${DCCA}_1$/W1 and RI	$0.907 \pm 0.009$	$0.76 \pm 0.01$
${DCCA}_0$/W1 and RI	$0.725 \pm 0.008$	$0.800 \pm 0.009$

**Table 2:** Cross-correlation exponents considering the well W1 and River RI (Wavelet Filter 0.70).

Method/time-series	Region I	Region II
${DFA}_1$/Well 1 (W1) Wavelet fILter 0.70	$0.115 \pm 0.005$	$0.54 \pm 0.02$
${DFA}_1$/River	$1.44 \pm 0.02$	$1.11 \pm 0.02$
${DFA}_0$/Well 1 (W1) Wavelet filter 0.70	$0.50 \pm 0.02$	$0.976 \pm 0.003$
${DFA}_0$/River	$0.867 \pm 0.007$	$0.56 \pm 0.02$
${DCCA}_1$/W1 (Wavelet filter 0.70) and RI	$0.915 \pm 0.007$	$0.939 \pm 0.008$
${DCCA}_0$/W1 (Wavelet filter 0.70) and RI	$0.768 \pm 0.008$	$0.83 \pm 0.01$
${DFA}_1$/Well 8 (W8) Wavelet filter 0.70	$0.114 \pm 0.005$	$0.74 \pm 0.02$
${DFA}_1$/Well 1 (W1)	$0.145 \pm 0.008$	$0.75 \pm 0.01$
${DFA}_0$/Well 8 (W8) Wavelet filter 0.70	$0.44 \pm 0.01$	$0.993 \pm 0.001$
${DFA}_0$/Well 1 (W1)	$0.39 \pm 0.01$	$0.999 \pm 0.002$

### Groundwater well-log deep-forecasting

**Figure 4:**
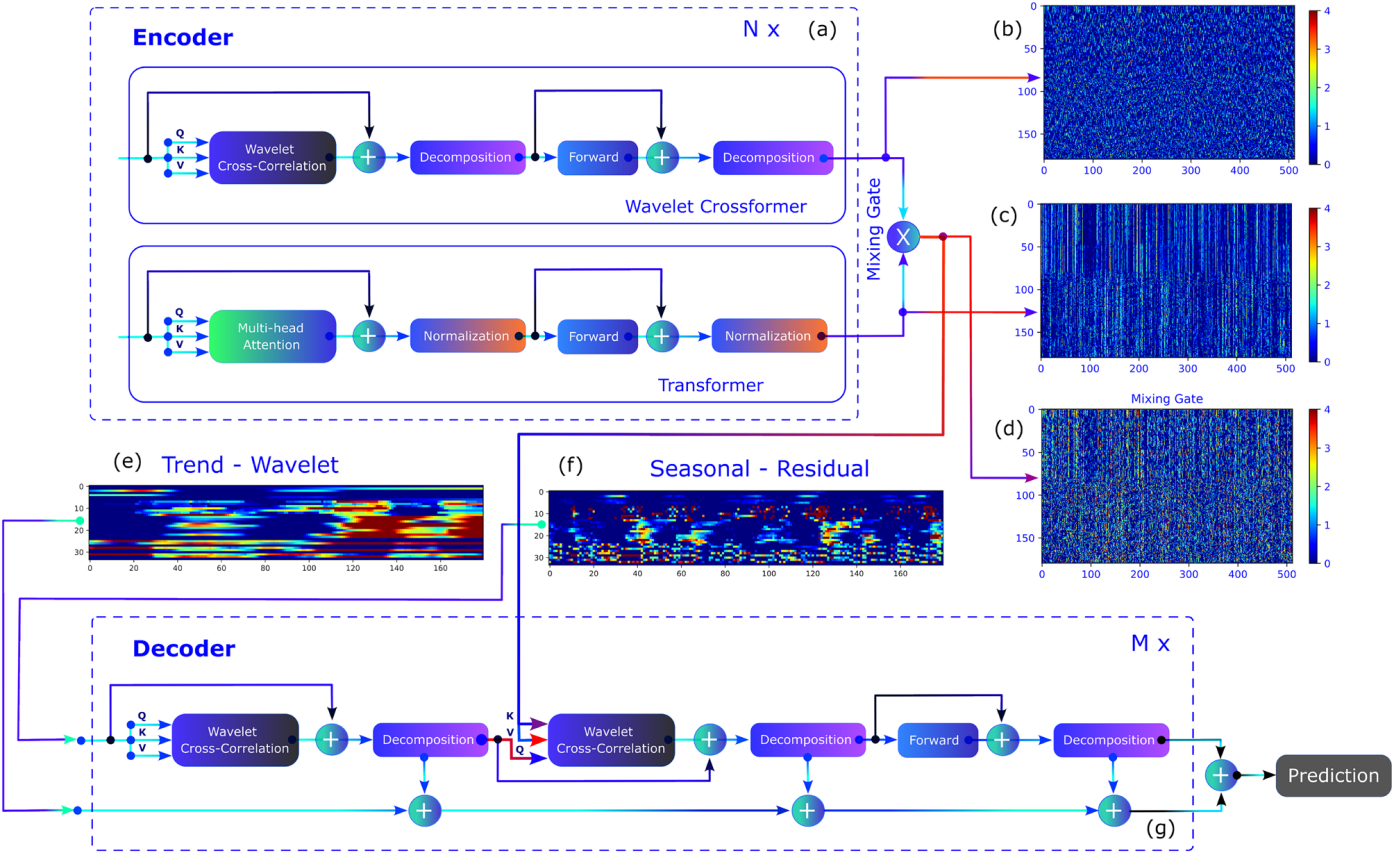
Wavelet Gated Multiformer with (**a**) Encoder, (**b**) random batch output signal from Wavelet Crossformer and (**c**) Transformer, and (**d**) signal after mixing gate. The Encoder’s output (**e**) wavelet cyclical trend, (**f**) seasonal trend, and (**g**) Decoder with inner cross-correlation blocks.

We have also predicted the most probable time series for a length *I* given an input signal *L* and a multivariate dataset obtained from multiple stations. We have proposed the Wavelet Gated Multiformer (details in the “Methods” section), which introduces a wavelet decomposition block for multivariate time-series forecasting inside the encoder. This approach simultaneously employs the advantages of past information from multiple sub-encoders through a mixing gate $\oplus$, as shown in Fig. 4. This procedure carries a combination of Transformer-based techniques since the output from the encoder has information regarding the relationship between scattered points (point-wise dependencies discovery) from the self-attention family^50,57^, aggregation of selected points by dot-product, and accumulation of similar sub-series from different periods through the time delay block (Wavelet Crossformer). Our approach can also be extended for application in other fields since the deep model provides higher accuracy for some well-logs.

**Table 3:** Time series forecasting results for wells W1–W8 with a predictive length of 30 and 60 days.

Well		Crossformer		Transformer		Autoformer		Informer	
Metric		MSE	MAE	MSE	MAE	MSE	MAE	MSE	MAE
W1	30	0.00925	0.07237	0.34608	0.52766	0.00987	0.08355	0.70871	0.80611
	60	0.00788	0.06840	0.27607	0.48360	0.01371	0.09950	0.85244	0.89560
W2	30	0.52115	0.61905	0.06888	0.20957	0.52275	0.67489	0.09626	0.26674
	60	0.46397	0.59317	0.09144	0.25529	0.74217	0.83077	0.10765	0.27641
W3	30	0.77759	0.82393	0.15215	0.33716	0.11117	0.27986	0.34037	0.55363
	60	0.61682	0.66926	0,19980	0.39619	0.11153	0.27952	0.33302	0.53747
W4	30	1.04818	0.82515	0.28464	0.39411	0.50697	0.53996	0.30920	0.43256
	60	1.29363	0.90303	0.24663	0.43913	0.36481	0.45908	0.58123	0,66646
W5	30	0.17268	0.36590	0.13576	0.32643	0.30318	0.52543	0.11061	0.27344
	60	0.23629	0.41187	0.14203	0.32898	0.46962	0.66320	0.07404	0.22836
W6	30	0.44984	0.53776	0.29903	0.47785	0.36899	0.48609	0.17024	0.33234
	60	0.44194	0.53514	0.23976	0.40862	0.22982	0.38238	0.20286	0.36610
W7	30	1.29190	0.98086	2.24612	1.46903	0.41498	0.54960	3.99748	1.97288
	60	–	–	2.78741	1.64702	0.15280	0.31165	4.86198	2.17386
W8	30	0.43957	0.50853	0.61219	0.74156	0.45841	0.52169	0.42389	0.60369
	60	0.48149	0.54576	0.58423	0.74164	0.54797	0.57954	0.52142	0.67114

**Figure 5:**
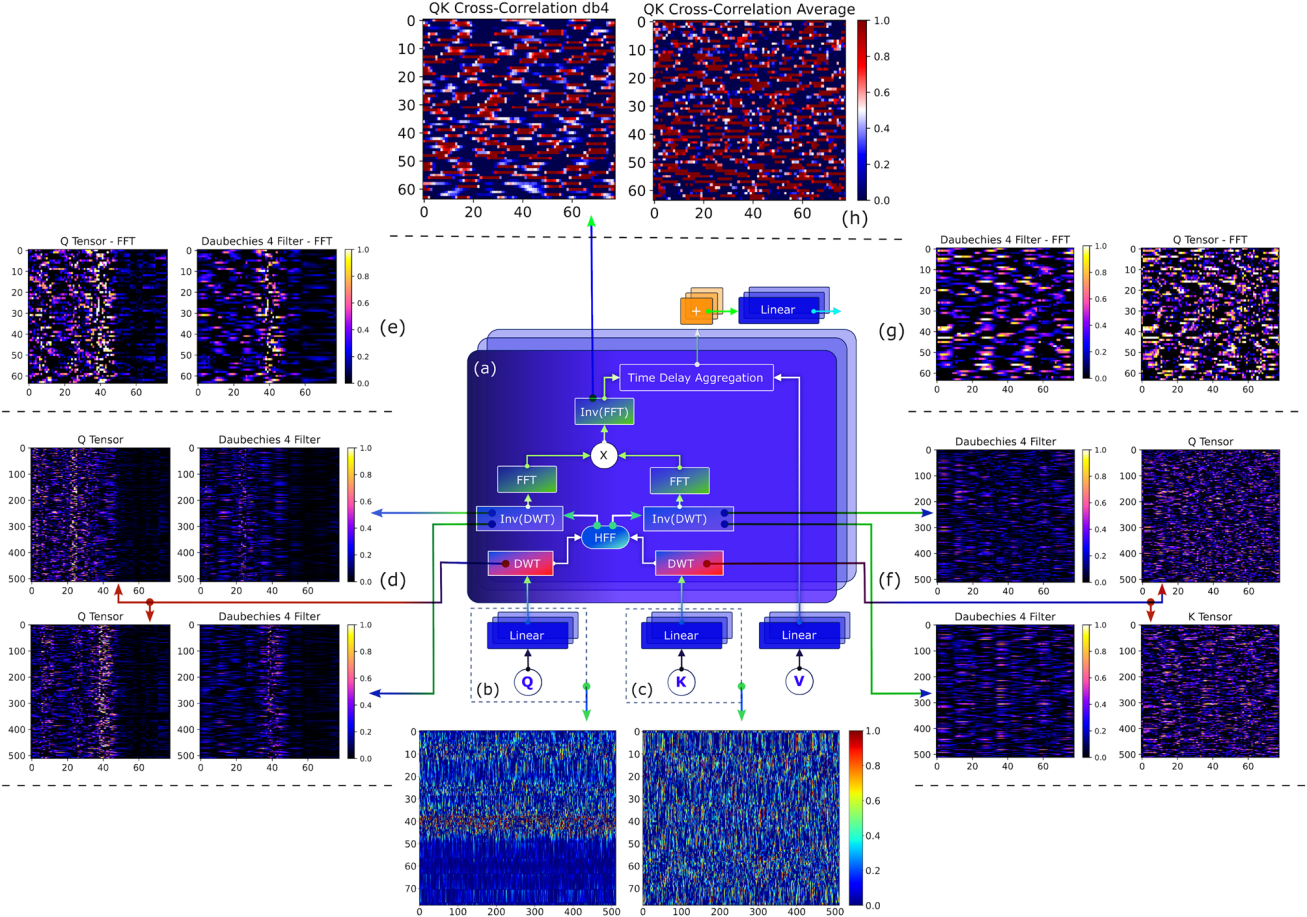
Wavelet Cross-correlation Block with (**a**) layer’s internal operations and self-attention input for (**b**) Queries and (**c**) Keys (K). Random batch tensor before and after denoising with wavelet db4 filter for (**d**, **e**) Queries and (**f**, **g**) Keys, respectively. (**h**) Represent a random batch for the Q-K cross-correlation using an average filter and wavelet db4.

Figure 4 shows the decomposed signal into trend-cyclical (long-term progression) and seasonal parts to understand the temporal patterns during the forecasting. The Wavelet Crossformer progressively uses a decomposition block similar to the Autoformer to pull the long-term stationary trend from intermediate predictions. However, instead of moving the average, we denoise the signal with a low-pass wavelet filter based on Daubechies 4 to concentrate on the long-term trends. Removing high-frequency noise has proven to be an essential factor for trend feature extraction. We have performed multiple experiments with eight wells by training a sequence of 180 time steps (days) to predict the next 30 and 60 time steps (days). The results are summarized in Table 3. The proposed Wavelet Gated Multiformer was also compared against the vanilla Transformer^45^, Autoformer^56^, and Informer^57^ using two different metrics: (i) Mean Squared Error (MSE), and (ii) Mean Absolute Error (MAE). The Wavelet Gated Multiformer, which takes advantage of the Cross-correlation Blocks, was considerably ahead of different transformers in wells W1 and W8. However, we have obtained mixed performance for the other six wells without a dominant architecture concerning the metrics MSE and MAE (Fig. 5).

**Figure 6:**
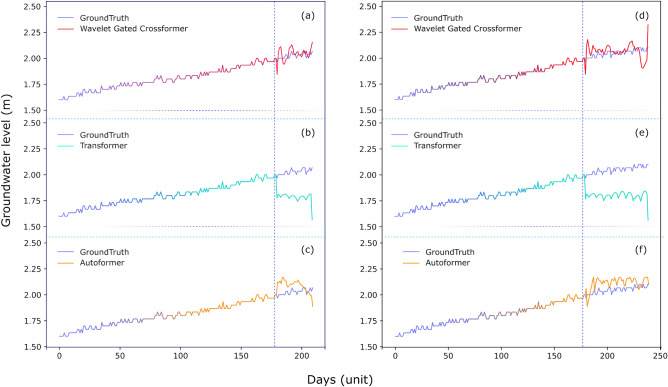
Predictions of 30 and 60 days for groundwater well P1 with (**a**, **d**) Wavelet Gated Multiformer, (**b**, **e**) Transformer, and (**c**, **f**) Autoformer with input size of 180 days, respectively.

**Figure 7:**
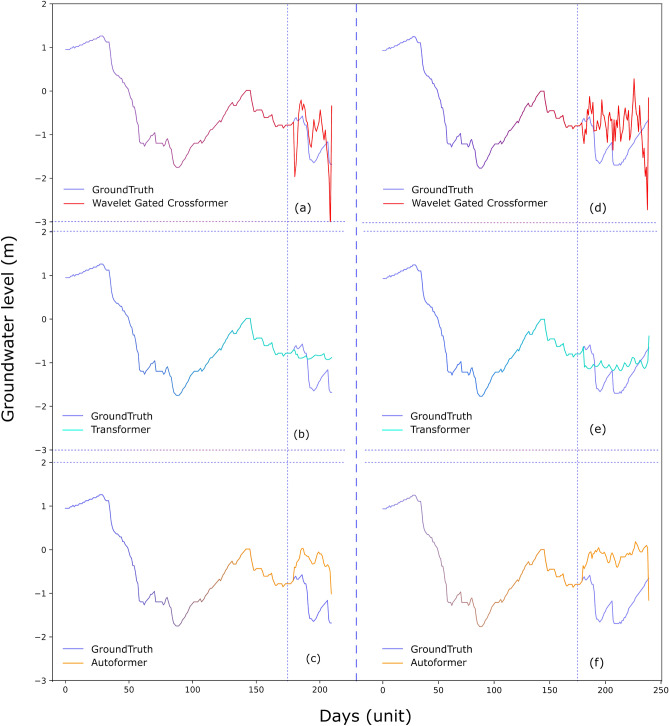
Predictions of 30 and 60 days for groundwater well P4 with (**a**, **d**) Wavelet Gated Multiformer, (**b**, **e**) Transformer, and (**c**, **f**) Autoformer with input size of 180 days, respectively.

Comparing the predictions of these three transformer-like architectures can also shed light on their capabilities. Figure 6 shows the predictions for the well W1, with the Wavelet Gated Crossformer providing better performance (MSE and MAE) for an input length of 180 days and prediction length of 30 days (left) and 60 days (right). However, the Transformer’s prediction falls entirely out of the signal trend, and the Autoformer fails to stabilize the signal over the curve. The Wavelet Gated Crossformer has shown higher variance, which can be induced by the selected Daubechies 4 wavelet, which provides the decomposed signal in trend and seasonal parts. Figure 7 shows the predictions on the well W4, where the Wavelet Gated Crossformer had the worst performance compared to the other transformers. However, the Wavelet Gated Crossformer still captures the general trend in its prediction for both 30 days and 60 days prediction lengths.

Figure 4a highlights the schematics of our encoder, composed of parallel N layers of multiple sub-encoders. The output of each sub-encoder is then mixed through a linear combination represented by the mixing gate $\oplus$. Figure 4b–d show the output Wavelet Crossformer sub-encoder, the Transformer sub encoder, and the result of utilizing past information from multiple sub-encoders through the Mixing Gate $\oplus$, respectively. The Wavelet Crossformer uses progressive decomposition blocks similar to the Autoformer to deal with long-term stationery trends with a low-pass wavelet filter based on Daubechies 4 to denoise the output signals and extract long-term trends. High-frequency signal denoising has proven to be an essential step for feature extraction inside the blocks. Furthermore, the Mixing Gate combines the trending output of the Transformer embeds information concerning the relationship between scattered points (point-wise dependencies discovery) from the self-attention family^50,57^ and aggregation of selected points by dot-product, and the accumulation of similar sub-series from different periods through the time delay block (Wavelet Crossformer). Figure 4d clearly shows the weighted characteristics of both sub-encoders after the Mixing Gate. 4e, f show the decomposed signal into trend-cyclical (long-term progression) and seasonal parts to understand the temporal patterns. Our proposed encoder and its operations are detailed in the “Methods” section.

Our Decoder is shown in Fig. 4g, detailed in the “Methods” section, which has M layers and two different inputs, a trend cyclical and seasonal (residual) derived from the encoder input. From Fig. 4e, the cyclical trend part, one can see that the signal has a lower frequency (long-term tendencies) as compared to the signals in Fig. 4f, where fast oscillations are dominant. Our Decoder also includes the wavelet denoising through the Wavelet Decomposition Block. The Decoder extracts the trend from the hidden variables and allows the Wavelet Gated Multiformer to refine the trend prediction by denoising the signals with wavelet filters for period-based pattern recognition inside the Wavelet Auto-Correlation block.

The cross-correlation block from Wavelet Gated Crosformer significantly improves the removal of high-frequency noise, as seen in Fig. 5. Before effective cross-correlation, both *Q* and *V*, as seen in Fig. 5b, c, are filtered with Wavelet Shrinkage Denoising using Daubechies 4 wavelet with a *soft* threshold for coefficients estimations. Fig. 5d shows the effect of wavelet filtering in two random batches with *Q* tensor samples. The high-frequency noise content is removed from the original signal under a specific threshold. The same outcomes are observed for *K* in Fig. 5f. Figure 5e, g, with the signal in the frequency domain, also supports the enhancement in quality provided by wavelet filtering. Finally, the outcomes of *QK* cross-correlation with and without wavelet denoise are compared in Fig. 5h, with explicit attenuation of white noise on the former. This process provides effective results once these signals are cross-correlated and better estimations for the TopK values used in time-delay aggregation of the value component *V*.

The deep forecasting models were trained with a multivariate dataset of rain, climate (atmospheric pressure, temperature, and humidity sensors), river, and well-log data that comprises 4 years of continuum daily measures from 34 sensors with a total of 49708 data points. We have calibrated the dataset with standardization (mean equal to zero and standard deviation equal to one). The input has a sequence length of 180 and a prediction length of 30 and 60 days. We have split the dataset with $70\%$ for training, $10\%$ for validation, and $20\%$ for testing purposes. We have also fine-tuned and adjusted the following parameters for the validation: (i) input sequence length of 180, (ii) batch size of 32, (iii) encoder input and output size of 34, and (iv) 15 training epochs. For each epoch, we have assessed the loss function concerning the training, validation, and testing to understand possible bias-variance tradeoffs and overfitting issues. Furthermore, we have also used a dropout of 0.05 to improve the convergence. These models were also trained with L2 loss function, ADAM^70^ optimizer with a learning rate of $10^{ - 4}$ and batch sizes 32 for all experiments. The training process stopped within 15 epochs. All experiments were implemented in PyTorch^71^ and executed on multiple single GPUs, NVIDIA A100, with 40 GB VRAM each. The hyper-parameter threshold for the wavelet db4 was fixed at 0.5, and the mixing gate coefficient for the Transformer was set at 0.4. The Wavelet Gated Multiformer contains two encoder layers with two sub-encoders in parallel and one decoder layer.

## Discussion

We have explored the MF-DCCHM to discover zero-order (around the mean) and first-order (around the first-degree polynomial) cyclical patterns and trends among all pairs of signals in our dataset. These results showed the local and global behavior related to dependence for all pairs of physical quantities analyzed. The main result can be verified in the heatmaps from Figs. 2 and 3 with the attenuation variance after filtering the trend, employing a sliding window, and obtaining, for all scales, all intensity levels related to correlation coefficients. Therefore, the heatmap was built with much more information when compared with previous works^1,2^. Furthermore, the heatmaps provided seasonal trends and cycles of 90 and 180 days, guiding our decision to use the deep-forecasting models with 30 and 60 days prediction lengths. It is essential to highlight that applying the Daubechies (db4) wavelet filters can reduce the noise and increase the magnitude of low-frequency signals to uncover cyclical trends. We have also shown that using the low-pass wavelet-based filters allows one to denoise the fluctuations and, in the case of well W8, to reduce the multifractal exponents for less than one. We have also found high cross-correlated cyclical patterns indicating a signature compatible with weather anomaly. In this case, the air can saturate under high relative humidity, and at a particular temperature, the air cannot hold water content, leading to clouds and further precipitation, also known as the dew point. Furthermore, we have also noticed a weakening of the detrended cross-correlation coefficients when considering different locations. Figure 2f shows the MF-DCCHM for temperature and humidity at stations T1 and H1. The average attenuates for different distances. However, the period for events is of approximately 6 months.

Regarding the multivariate time-series deep-model forecasting approach, Fig. 4 shows our proposed Wavelet Gated Crossformer, which takes advantage of multiple sub-encoders with the mixing gates. Our model carries information regarding all parallel sub-encoders, and it can be generalized for multiple sub-encoders and decoders, possibly extrapolating the capacities of any single transformer-like architecture. Additionally, wavelet decomposition blocks provide another route to separate signals in trend and seasonal parts as the moving average from the Autoformer suffers from the border effect at the beginning and later parts of the curve. Furthermore, Wavelet Shrinkage Denoising can overcome this limitation on capturing trend signals by extracting wavelet coefficients associated with higher frequencies. The proposed architectures also implement a new Wavelet Cross-Correlation Block that employs wavelets to denoise the signal before the inner QK cross-correlation. The improved signal quality concatenates the cross-correlation and provides better estimations for the time delay aggregation. The Wavelet Gated Multiformer has provided better results in two of the eight wells when compared to the Autoformer and Transformer, reducing Mean Absolute Error (MAE) by 31.26 % compared to the second-best performing transformer-like models evaluated. Nevertheless, considering only predictions trends, our approach efficiently captures long-term patterns and has the potential for application in other fields.

Deep learning has been successfully used to uncover subtle and hidden patterns in time series data. Deep Learning models have been used to understand traffic flow and congestion (time series) and have shown promise in time-series forecasting. The most recent deep-forecasting models can outperform statistical methods for large multivariate datasets. Transformers are used to perform natural language processing (NLP) tasks, with several architectures for long-term forecastings, such as FEDformer, PatchTST, Informer, Autoformer, and Transformer. Our article provides a new deep-forecasting model to predict groundwater level from a multivariate dataset which includes rain, climate (atmospheric pressure, temperature, and humidity sensors), river, and well data and comprises 4 years of continuum daily measures from 34 sensors (or 49708 data points). However, performance is a key limitation of Wavelet Gated Multiformer because of its parallel encoder structures and the additional wavelet denoise shrinkage. Future works could focus on computational costs, benefit from the new approach, and explore combinations of different transformers-like models. Furthermore, research prospects in this field could investigate the potential of the Wavelet Gated Multiformer for Time Series Forecasting using open-source benchmarks such as the Electricity Transformer Temperature (ETT), a crucial indicator in long-term electric power deployment.

## Methods

### Encoder

The encoder shown in Fig. 4 is decomposed into two sub encoders (Wavelet Crossformer and Transformer) and mixed through a linear combination represented by the mixing gate $\oplus$ after N blocks. Inside the sub-encoder Wavelet Crossformer, the trending part of the signal is discarded at the Wavelet Decomposition block, while the seasonal part remains. The input of the sub-encoder is composed of the past input-I time steps $X_{en} \in R^{I,d}$. Let $\Omega$ represent the Wavelet-Correlation Block and $\Gamma$ the Wavelet Decomposition Block, the overall equation of the *l*-th Crossformer sub encoder layer can be expressed as follows:

1 $$\begin{aligned} \begin{aligned} S_{en}^{l,1} , T_{en}^{l,1}&= \Gamma (\Omega (\chi _{en}^{l-1}) + {\chi }_{en}^{l-1} ) \\ S_{en}^{l,2}, T_{en}^{l,2}&= \Gamma (FeedForward(S_{en}^{l,1}) + S_{en}^{l,1} ) \end{aligned} \end{aligned}$$

where $T_{en}^{l,1}$ represents a removed trend signal from the Crossformer, $\chi _{i}^l = S_{en}^{l,2}$ such that $l=\{1,.., N\}$ represents the outcome of the l-th sub-encoder layer and $X_{en}^{0}$ is the embedded $X_{en}$. The seasonal component after the first or second $k=\{1,2\}$ series wavelet decomposition block in the l-th sub-encoder layer is represented by $S_{en}^{l,k}$.

On the other hand, in the Transformer encoder, there is no Wavelet Decomposition, and the signal is fully considered. Its equations have the following operations:

2 $$\begin{aligned} \begin{aligned} A_{en}^{l,1}&= Norm(MultiHeadAttention(\chi _{en}^{l-1}) + \chi _{en}^{l-1}) \\ A_{en}^{l,2}&= Norm(FeedForward(A_{en}^{l,1}) + A_{en}^{l,1}) \end{aligned} \end{aligned}$$

where $\chi _{j}^l = A_{en}^{l,2}$ represents the outcome of the l-th Transformer layer. The final operation in the mixing gate $\chi _{en}^l = \chi _{i}^l \oplus \chi _{j}^l$ can be expressed as:

3 $$\begin{aligned} G_{en} = \alpha * S_{en}^{N,2} + \beta * A_{en}^{N,2} \end{aligned}$$

and $G_{en}$ is the Encoder output trend, composed of a linear combination of parallel sub-encoders. The model is not restricted to only two sub-encoders as it can be generalized for a linear combination of multiple encoders.

#### Decoder

In this model, the decoder input is divided in two: a seasonal part $\chi _{des} \in R^{(\frac{I}{2}+O)xd}$ and a cyclical trend part $\chi _{det} \in R^{(\frac{I}{2}+O)xd}$, each of which is formulated by a specific rule. $\chi _{des}$ is the concatenation of the latter half of the seasonal part of the encoder’s input with *O* zeros, and $\chi _{det}$ is the concatenations of the latter half of the trend part of the encoder’s input with *O* values representing the average of the encoder’s input $\chi _{en}$. One can put it formally as:

4 $$\begin{aligned} &\chi _{ens},\chi _{ent} = \Gamma (\chi _{en \frac{I}{2}:I}) \\&\chi _{des} = Concat(\chi _{ens}, \chi _0) \\&\chi _{det} = Concat(\chi _{ent}, \chi _{Mean}) \end{aligned}$$

where $\chi _{ens}, \chi _{ent} \in R^{\frac{I}{2}xd}$ are the seasonal and trend parts of $\chi _{en}$, respectively, and $\chi _{0}, \chi _{Mean} \in R^{Oxd}$ represent placeholders filled with zero and the mean of $\chi _{en}$, respectively.

Figure 4 shows the decoder with two components: the trend-cyclical accumulated over the decoder and a seasonal part stacked in a series of blocks. We have introduced wavelet denoising in the Wavelet Decomposition Block to replace the original Series Decomposition block from Autoformer. Inside the decoder, information from the encoder is integrated as *K* and *V* on one of its Autocorrelation blocks with wavelet denoising. The decoder extracts the trend from the hidden variables and allows the Wavelet Gated Multiformer to refine the trend prediction by denoising the signals with wavelet filters for period-based pattern recognition inside the Wavelet Cross-Correlation block. The decoder has M layers and receives input $\chi _{en}^l$ from the encoder, and past information from the decoder, such that the l-th decoder layer can be represented by $\chi _{de}^l = Decoder(\chi _{de}^{l-1},\chi _{en}^N)$, where the internal operations can be described as:

5 $$\begin{aligned}&S_{de}^{l,1} , T_{de}^{l,1} = \Gamma ( \Omega (X_{de}^{l-1}) + X_{de}^{l-1} ) \\&S_{de}^{l,2}, T_{de}^{l,2} = \Gamma ( \Omega (S_{de}^{l,1},X_{en}^{N}) + S_{de}^{l,1} ) \\&S_{de}^{l,3}, T_{de}^{l,3} = \Gamma ( FeedForward(S_{de}^{l,2}) + S_{de}^{l,2} ) \\&T_{de}^{l} = T_{de}^{l-1} + W_{l,1} * T_{de}^{l,1} + W_{l,2} * T_{de}^{l,2} + W_{l,3} * T_{de}^{l,3} \end{aligned}$$

where $\chi _{de}^{l} = S_{de}^{l,3}, l \in \{1,...,M\}$ is the output of *l*-th decoder layer. $\chi _{de}^0$ is embedded from $\chi _{des}$ and $T_{de}^0 = \chi _{det}$. Therefore, the decoder’s prediction output is represented by the sum of two decomposed components $W_{S}*\chi _{de}^{M}+T_{de}^M$, where $W_{S}$ indicates the projection of seasonal components $X_{de}^M$ to the proper output dimension and *M* is the number of decoder layers. The variables $S_{de}^{l,3}$ and $T_{de}^{l,3}$ characterize the seasonal and trend-cyclical components for the first, second, and third wavelet-series decomposition block, respectively. The $W_{l,i}$ represents the projection for the trend $T_{de}^{l,i}$, where $i=\{1,2,3\}$ is directly associated with the first, second, and third decoder’s inner wavelet-series decomposition block.

### Wavelet decomposition block

#### Wavelet shrinkage denoising

Wavelets form a basis in the space of integrable square functions and, as other approaches like Fourier series, local polynomials, splines, and kernels, can be used to represent unknown functions. One advantage of wavelets over the other approaches is that they are *localized* in time, as this property substantially decreases the computational cost in the representation of the functions. The Wavelet Transform (WT) coefficients of a signal *x*(*t*) are obtained from the following equation:

6 $$\begin{aligned} X_w (a,b) = \frac{1}{|a|^{1/2}} \int \limits _{- \infty }^{+ \infty } x(t) \overline{\psi } (\frac{t-b}{a}) dt, \end{aligned}$$

where $\psi (t)$ is the continuous root wavelet scaled by a factor *a* and mapped with factor *b*. The continuous representation has infinite coefficients *a* and *b* mapped through the Continuous Wavelet Transform (CWT). However, due to the intense computational cost, the CWT is only feasible when considering discrete signals with limited coefficients. Instead, most applications use the Discrete Wavelet Transform (DWT). In DWT, the efficiency comes from the increase of *a* and *b* factors based on the power of two.

Some of these decomposed wavelet coefficients correspond to signal averages, and others are associated with details on the original signal. DWT coefficients are filtered with a threshold to denoise or recover the partial trend. Additionally, we apply an inverse wavelet transform to restore the filtered signal to the time domain, a process known as wavelet shrinkage denoising. Based on the methodology developed by Donoho and Johnstone^72^, we have employed *soft* threshold to estimate the wavelet coefficients of the trending signal.

### Wavelet decomposition

We have used the decomposition to cluster the signal in two subgroups representing a long-trend pattern with low frequency and a seasonal part with higher frequency and more fluctuations. Unlike Autoformer, which computes an average of a padded signal, we have proposed the low-pass filter based on wavelet decomposition, which removes the need for paddings. For a signal $\chi \in R^{L x d}$ the decomposition process is:

7 $$\begin{aligned} &T = WaveletDecomp(\chi , threshold) \\&S = \chi -T \end{aligned}$$

where *S* and *T* denote the seasonal and trend-cyclical signals, respectively, and the soft *threshold*^72^ is employed to filter the wavelet coefficients of the trending signal. For the sake of simplicity, the decomposition blocks were denoted as $S, T = \Gamma (\chi )$ in the previous sections.

### Wavelet cross-correlation block

The Wavelet Cross-correlation Block is an extension of the autocorrelation block introduced by the Autoformer architecture^56^. Figure 5 shows that, in Wavelet Cross-correlation Block, the components *Q* and *K* are denoised through wavelet shrinkage before the autocorrelation computation. The cross-correlation is done in three steps for better computation efficiency: (i) Fast Fourier Transform, (ii) multiplication in the frequency domain, and (iii) an inverse Fast Fourier Transform. The Wavelet cross-correlation block also discovers period-based dependencies by aggregating sub-series with the time-delay mechanism. The time delay aggregation is responsible for rolling the series based on selected time delay $\tau _1,...,\tau _n$. Therefore, the block aligns similar sub-series with a subsequent aggregation through the softmax normalized confidences. For a series *X* with length *L* and a single head, we can replace the self-attention mechanism considering that *Q*,*K*, and *V* values are obtained through the following operations:

8 $$\begin{aligned}&\tau _1, ..., \tau _k = argmax~Topk(R_{Q,K}(\tau )) \\&{\hat{R}}_{Q,K}(\tau _1),...,{\hat{R}}_{Q,K}(\tau _n) = SoftMax(R_{Q,K}(\tau _1),...,R_{Q,K}(\tau _n)) \\&CC(Q,K,V) = \sum _{i=1}^{k} Roll(V,\tau _i){\hat{R}}_{Q,K}(\tau _i) \end{aligned}$$

where $argmax~Topk(\cdot )$ represents the arguments of the *Topk* cross-correlations, $R_{Q, K}$ is cross-correlation, $Roll(V,\tau _i)$ represents the operation where *V* is shifted left by $\tau _i$, during which elements that are shifted beyond firs position are placed at last position. Furthermore, *K*, *V* are from the encoder $X^N_{en}$, which is resized to length *O*, and Q comes from the previous block of the decoder. Additionally, the multi-head would include the $Q_i,$, $K_i,$ and $V_i$, for $i=\{1,...,h\}$, where *h* represents the number of heads. The i-th head contains $Q_i, K_i,V_i \in R^{\frac{d_{model}}{h}}$, where $d_{model}$ represent the channels.

### Multifractal detrended cross-correlation heatmaps

The Multifractal Detrended Cross-Correlation Heatmaps were strictly computed according to Paulo et al.^62^. The procedure is to first obtain the long-range correlation through the Detrended Fluctuation Analysis (DFA)^69,73^. Additionally, we have estimated the Detrended Cross-Correlation Analysis (DCCA)^74^ for long-range cross-correlation memories. Finally, we compute the average over the fluctuations $F_X^2$ for all m-*ith* sliding boxes with different sizes *v* over the entire series, such that:

9 $$\begin{aligned} F_X^2 = \frac{1}{M_v} \sum _{m=1}^{M_v} f_X^2(m,v) \end{aligned}$$

$f_X^2(m,v)$ represents the variance and covariance of fluctuations, $M_v$ is the number of windows inside the entire series, and X characterizes the methods *DFA* and *DCCA*.

The computed variance and covariance can be characterized by a power-law $F_X \approx v^{\alpha }$ where *v* represents the box size. The scaling factor $\alpha$ was obtained by linearization $\log (F_X) X \log (v)$, where $\alpha = \lambda$ corresponds to the cross-correlation. Therefore, we can classify anti-persistent and persistent patterns based on the multifractal scaling factor $\alpha$. The trend can reverse shortly for $0<\alpha <0.5$. However, white noise could affect the integrated series and provide 0 cross-correlations for $\alpha = 0.5$. The integrated signal continues the previous trend for $0.5< \alpha <1.0$.

The final mapping quantifies the cross-correlation from a pair of non-stationary signals^75^ through its coefficients across multifractal regimes. We compute the Cross-Correlation Coefficient (CCC) as follows:

10 $$\begin{aligned} \sigma _{DCCA_i}(v,t) = \frac{F^2_{DCCA_i}(v)}{F_{DFA_i}(v) F'_{DFA_i}(v)}, \end{aligned}$$

where $i=\{0,1\}$, and $\sigma _{DCCA_i}(v,t)$ varies in the interval $-1 \le \sigma _{DCCA_i} \le 1$. The values of $\sigma _{DCCA_i}=\{1,0,-1\}$ represents maximum cross-correlation, no cross-correlation and anti-cross-correlation. This method allows one to observe potential cyclical patterns for different scales and multifractal regimes in one map and their consistency. The average sampling of DCCA over all possible windows of size *v* is shown on top of the cross-correlation heatmap.

### Supplementary Information

Supplementary Information.

## Data Availability

The datasets generated during and/or analysed during the current study are available from the corresponding author on reasonable request.
