# Supplementary material for: Wavelet gated multiformer for groundwater time series forecasting
Source: Sci Rep. 2023 Aug 5;13:12726. doi: 10.1038/s41598-023-39688-0 (PMC10404297; doi:10.1038/s41598-023-39688-0)
Supplement: Supplementary file 1 — Supplementary Information. [file 41598_2023_39688_MOESM1_ESM.pdf]

Wavelet Gated Multiformer for Groundwater Time Series Forecasting

Vitor Hugo Serravalle Reis Rodrigues [1], Paulo Roberto de Melo Barros Junior [2], Euler Bentes dos Santos Marinho [3], Jose Luis Lima de Jesus Silva [4, *]

[1] Geological Survey of Brazil - SGB, Avenida Ulysses Guimarães, 2862 Centro Administrativo da Bahia, Salvador-BA, 1649-026, Brazil
[2] Petrobras, Petróleo Brasileiro S.A., Av. República do Chile, nº 65 Centros, Rio de Janeiro, 20031-912, Brazil
[3] Federal University of Bahia, Center in Geophysics and Geosciences, Rua Barão de Jeremoabo, Ondina, Salvador-BA, 40210-630, Brazil
[4] Linköping University, Department of Computer and Information Science, Division of Artificial Intelligence and Integrated Computer Systems, Linköping, SE-581 83, Sweden

* jose.silva@liu.se

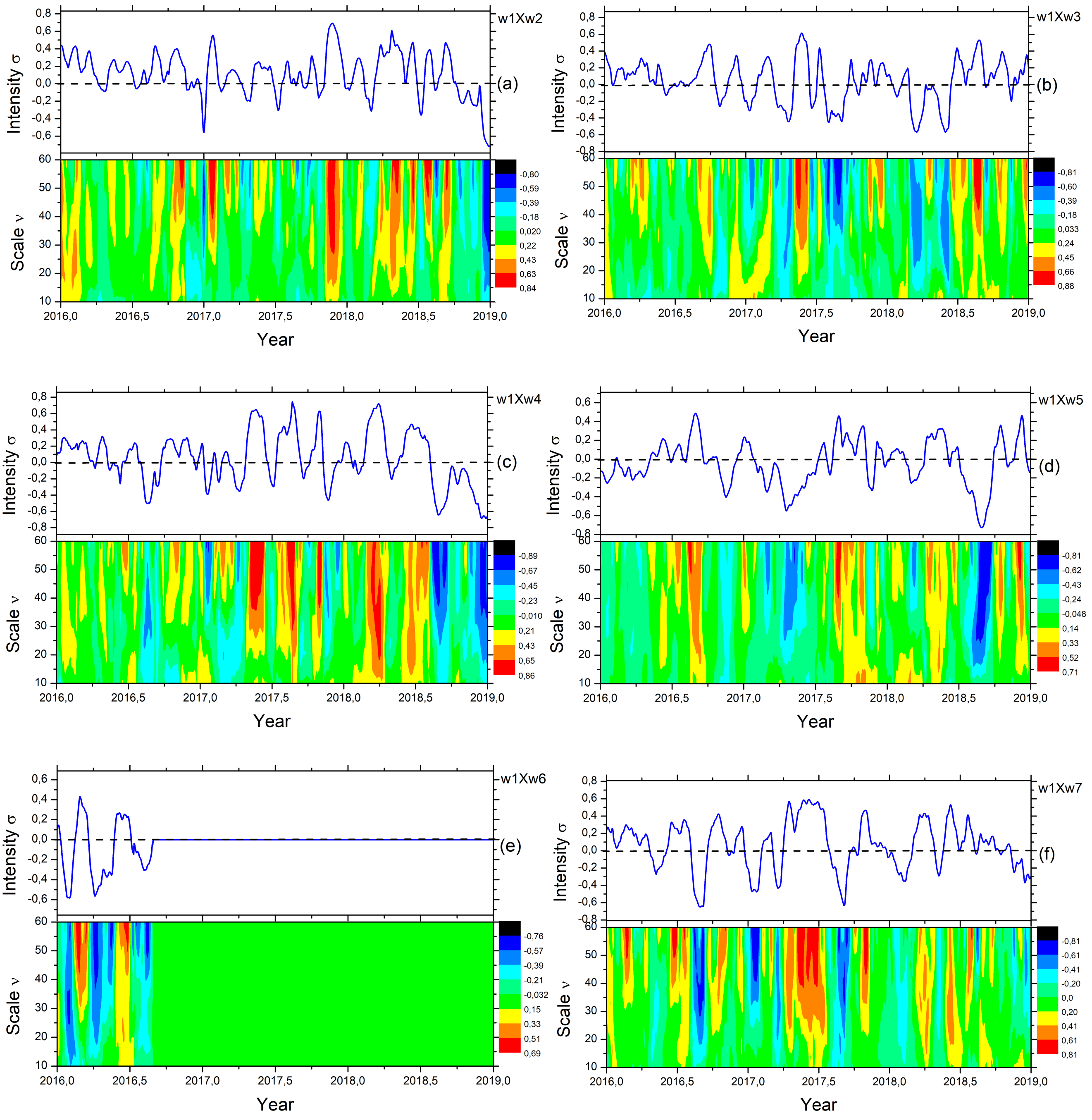

Figure S1. *Multifractal Detrended Cross-correlation Heatmaps between the attributes: (a) W1 and W2, (b) W1 and W3, (c) W1 and W4, (d) W1 and W5, (e) W1 and W6 (f) W1 and W7.*

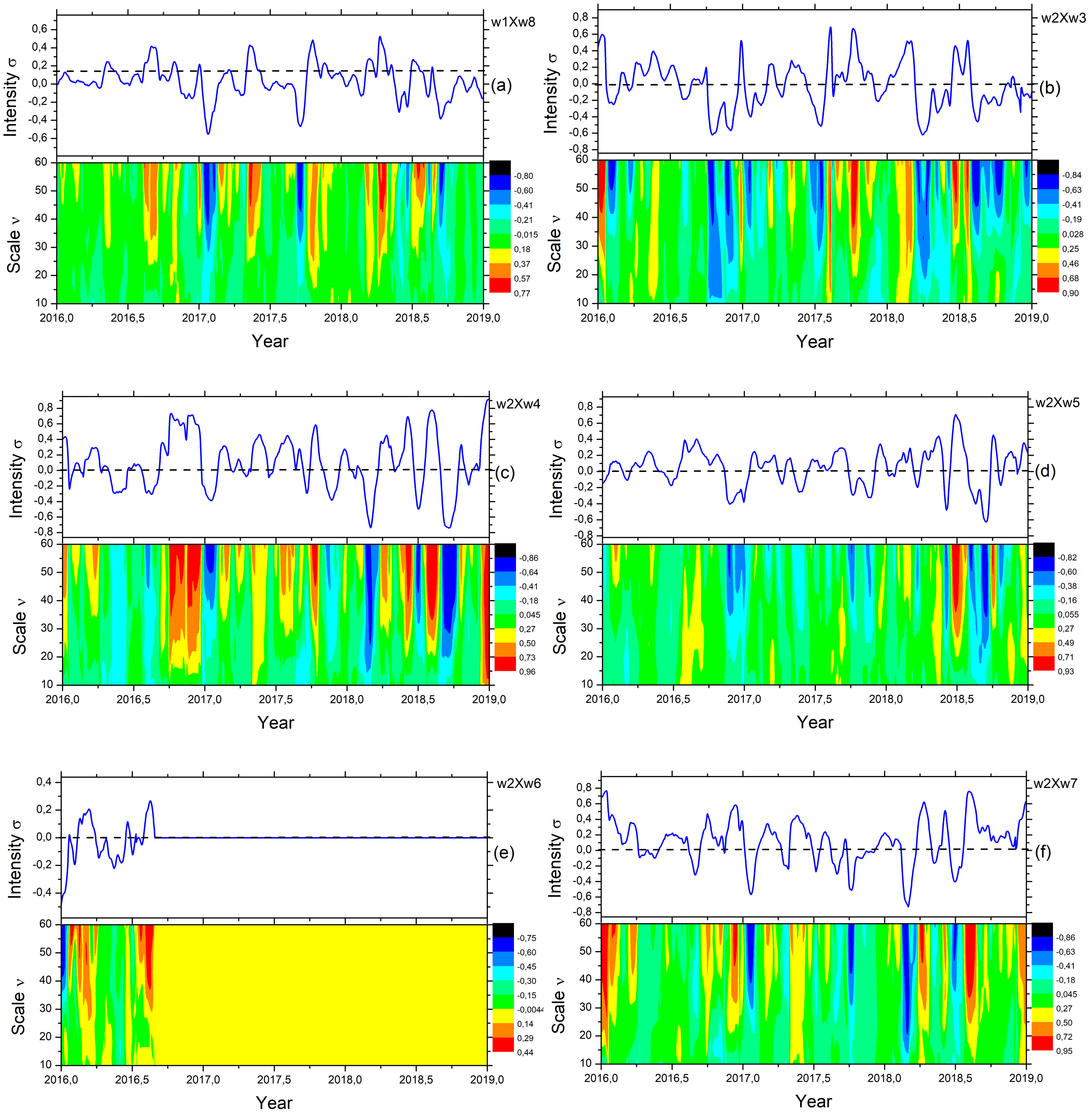

Figure S2. *Multifractal Detrended Cross-correlation Heatmaps between the attributes: (a) W1 and W8, (b) W2 and W3, (c) W2 and W4, (d) W2 and W5, (e) W2 and W6 (f) W2 and W7.*

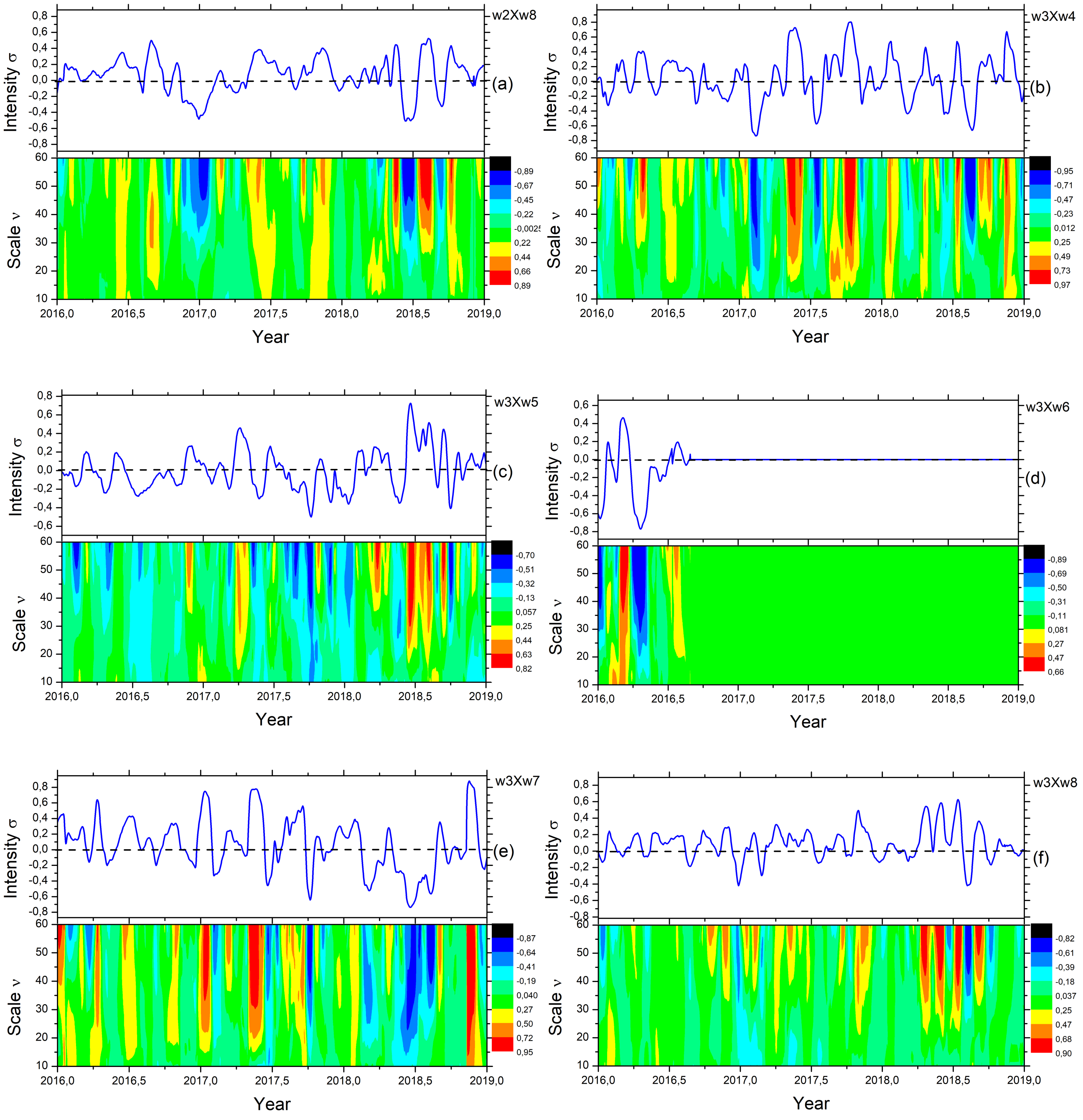

Figure S3. *Multifractal Detrended Cross-correlation Heatmaps between the attributes: (a) W2 and W8, (b) W3 and W4, (c) W3 and W5, (d) W3 and W6, (e) W3 and W7 (f) W3 and W8.*

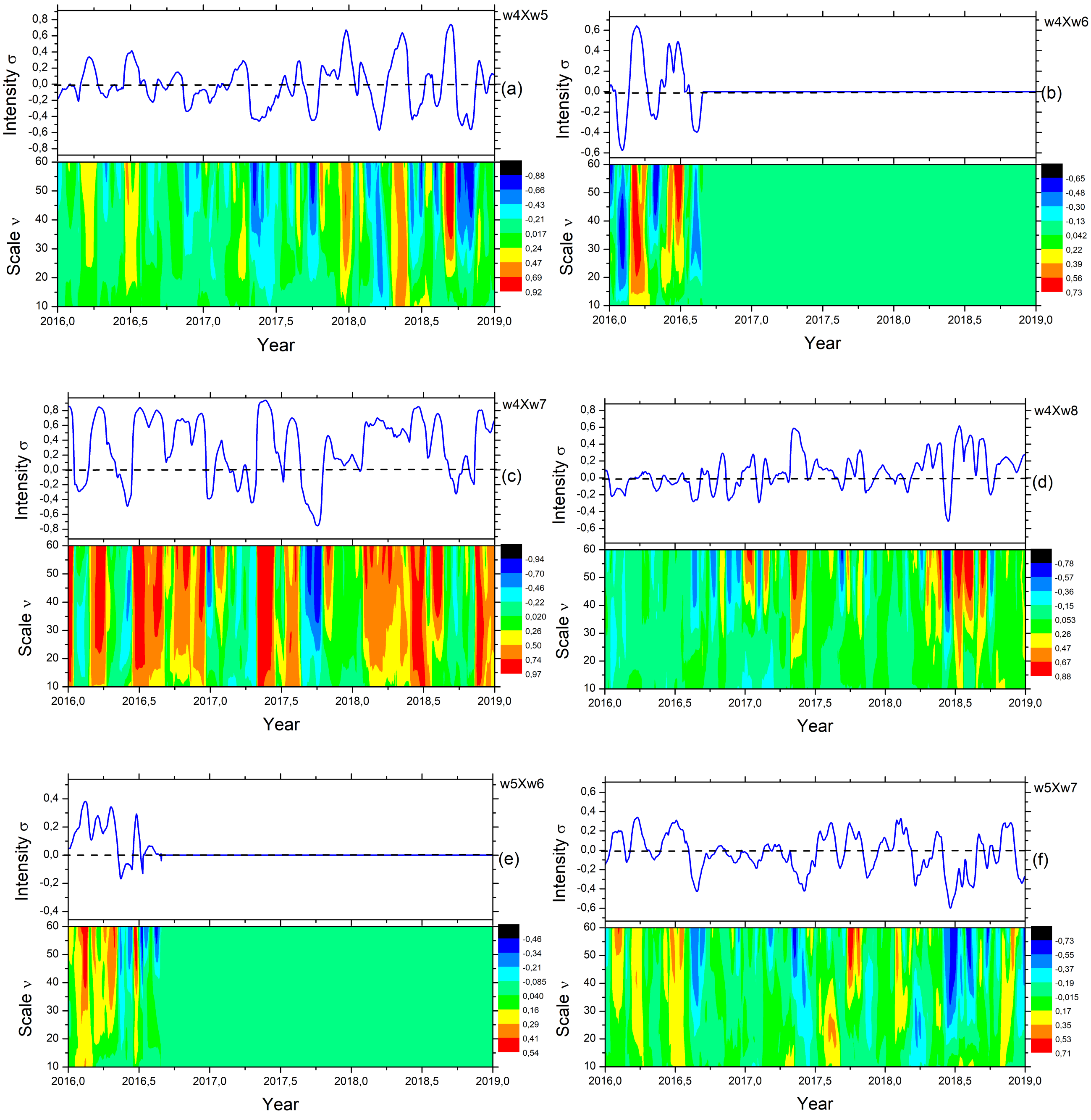

Figure S4. *Multifractal Detrended Cross-correlation Heatmaps between the attributes: (a) W4 and W5, (b) W4 and W6, (c) W4 and W7, (d) W4 and W8, (e) W5 and W6 (f) W5 and W7.*

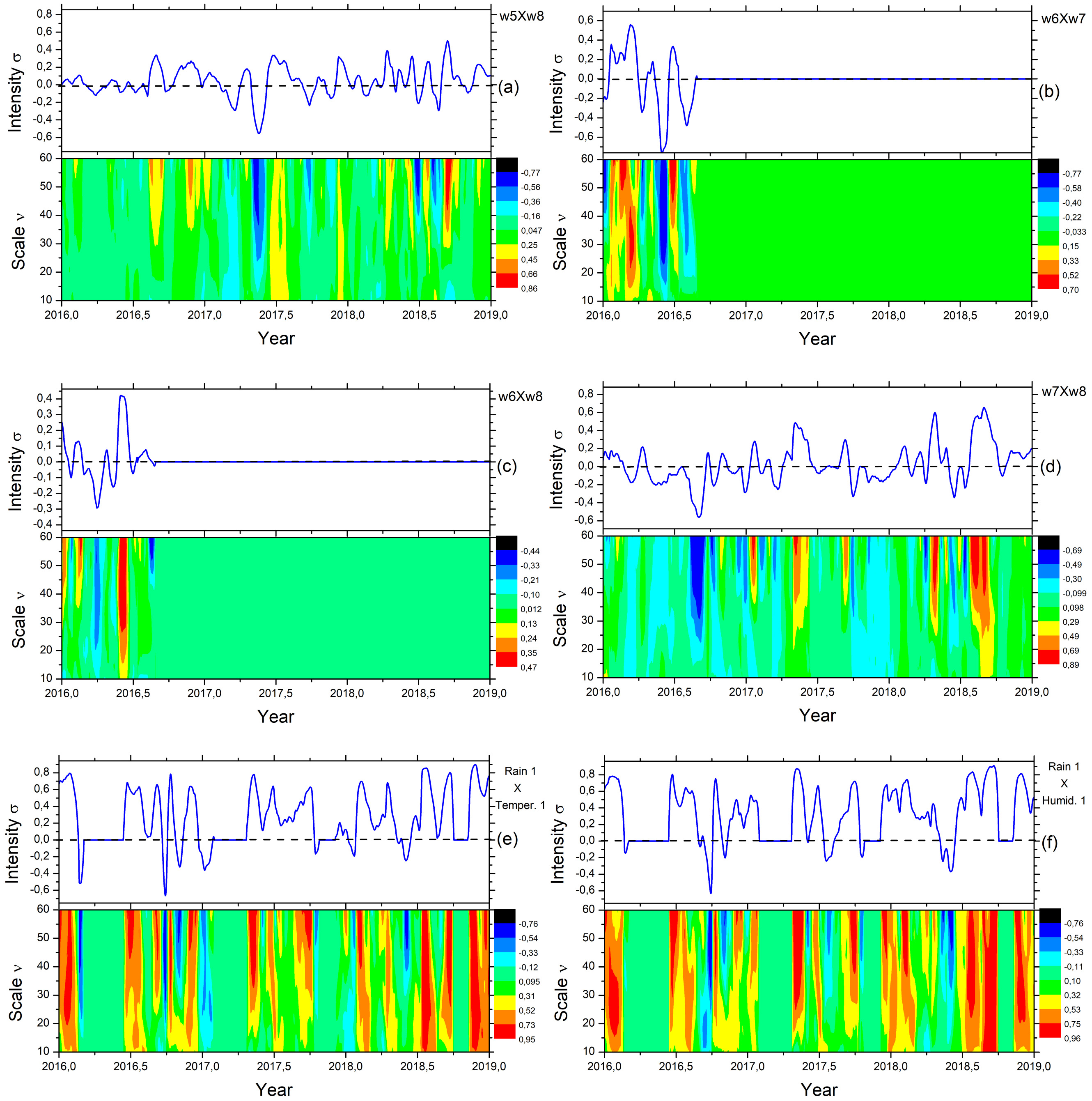

Figure S5. *Multifractal Detrended Cross-correlation Heatmaps between the attributes: (a) W5 and W8, (b) W6 and W7, (c) W6 and W8, (d) W7 and W8, (e) R01 and T01 (f) R01 and H01.*

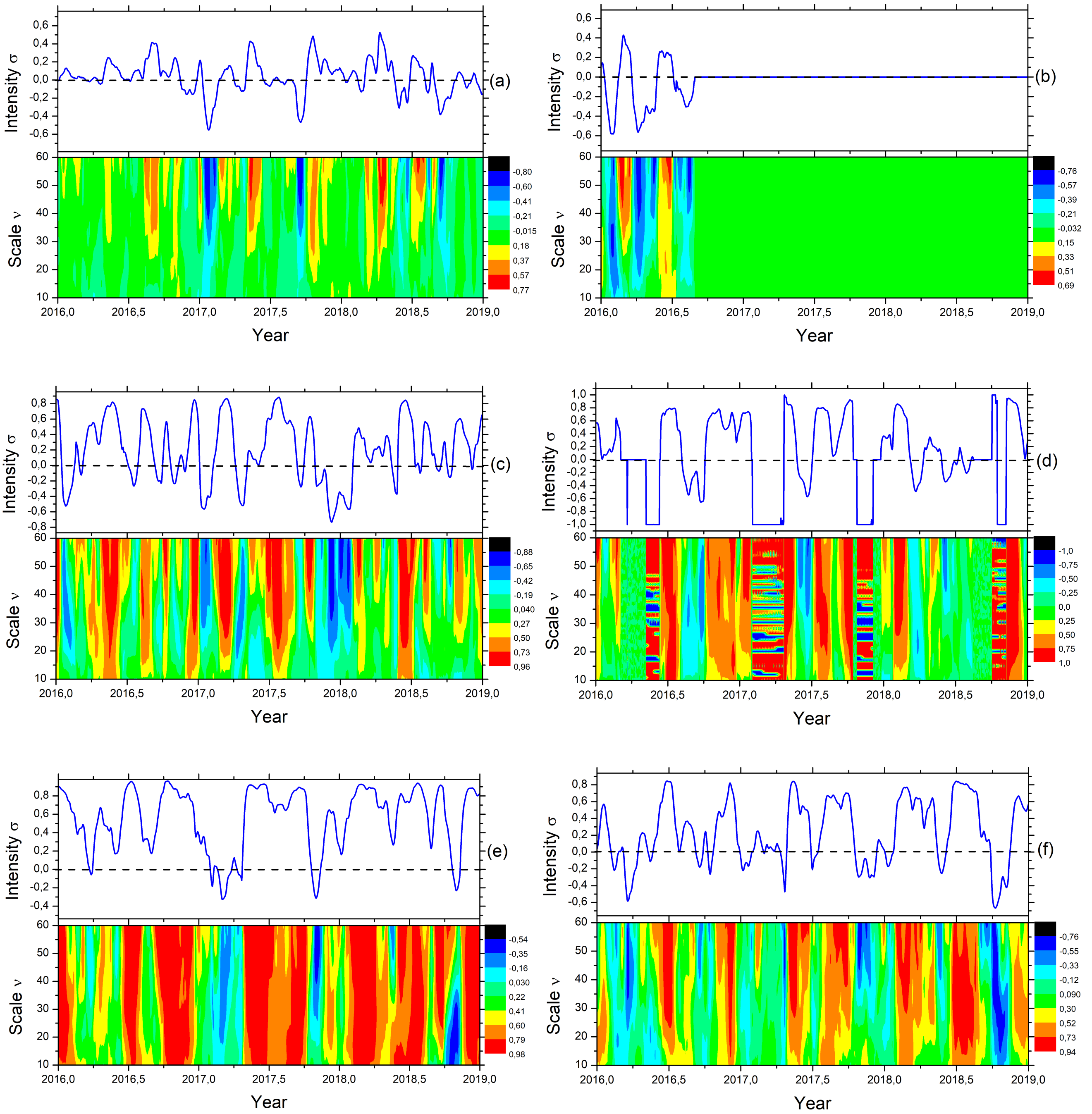

Figure S6. *Multifractal Detrended Cross-correlation Heatmaps between the attributes: (a) W1 and W8, (b) W1 and W6, (c) RI2 and RI10, (d) R01 and R04, (e) T01 and H01 (f) T01 and H02.*

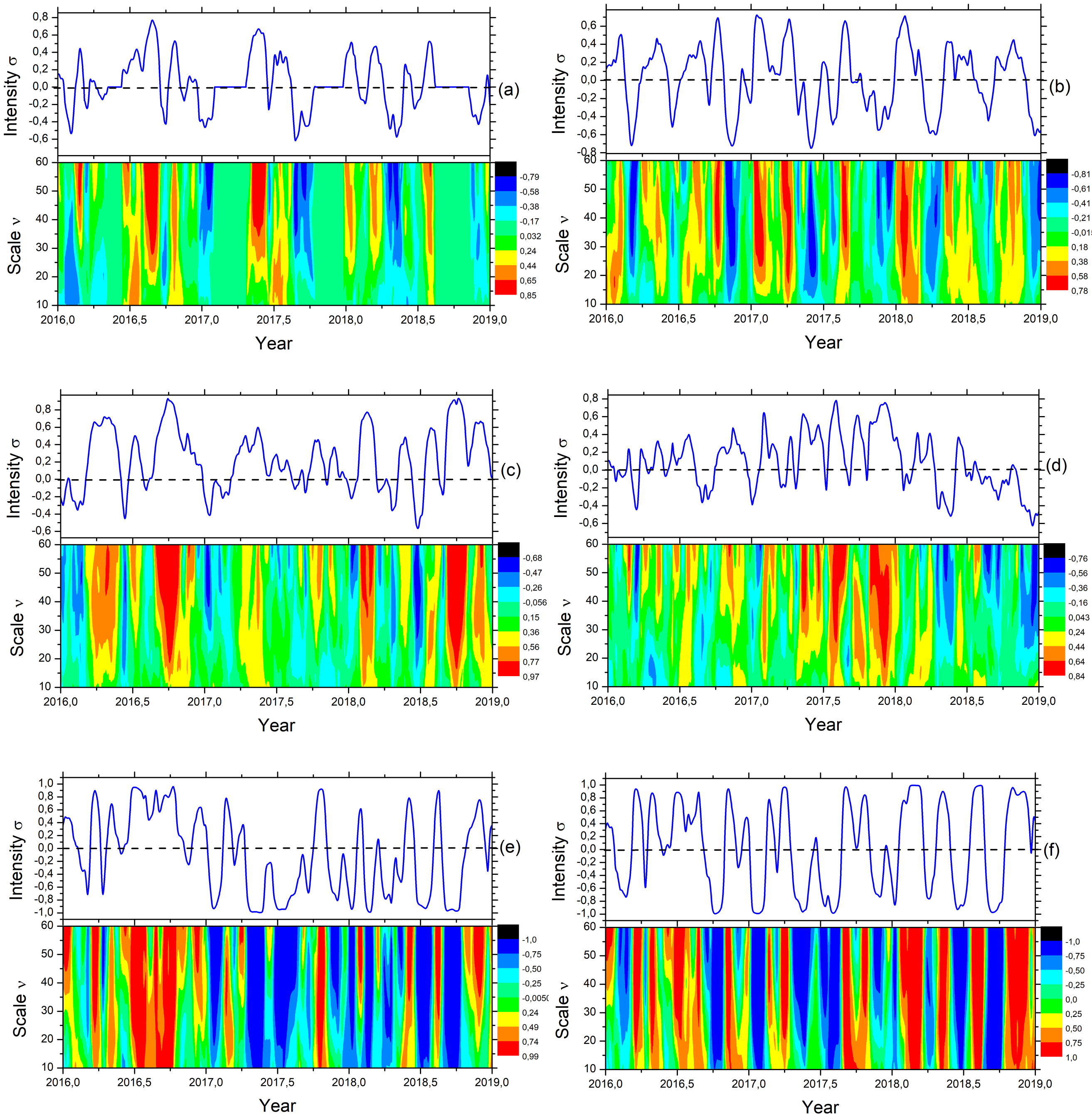

Figure S7. *Multifractal Detrended Cross-correlation Heatmaps between the attributes: (a) W01 and R04, (b) W01 and RI10, (c) P01 and T01, (d) W01 (Simple Filter) and RI, (e) W01 (Wavelet Filter 0.35) and RI and (f) W01 (Wavelet Filter 0.70) and RI.*

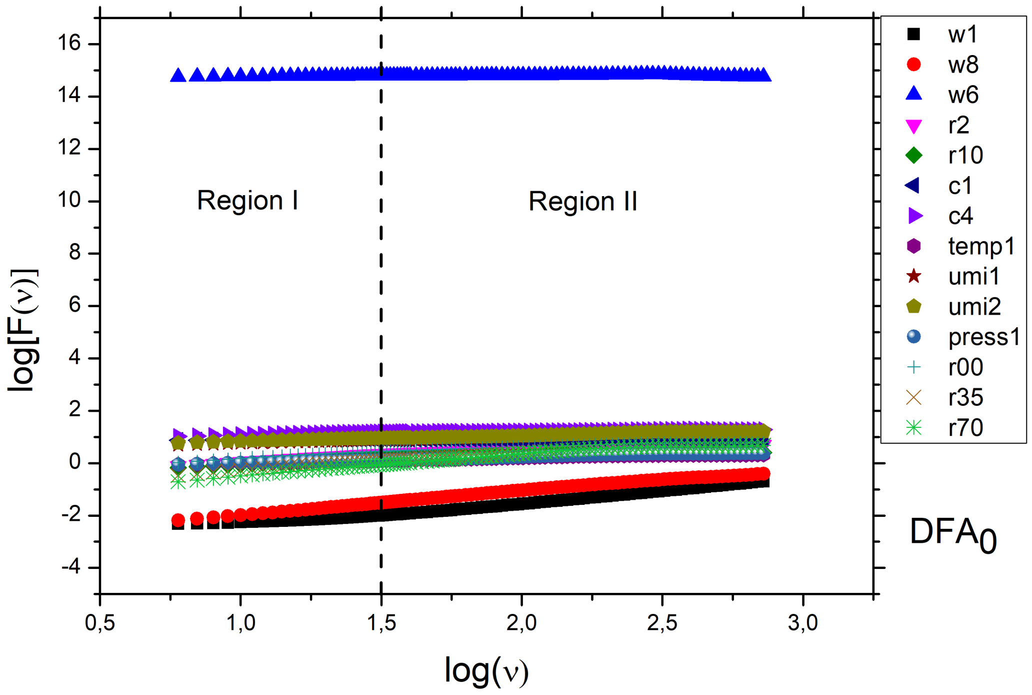

Figure S8. Detrended Fluctuation analysis around the average. The colors indicate the attributes. Each RI indicates stations summed and weighted by distances to the W1.

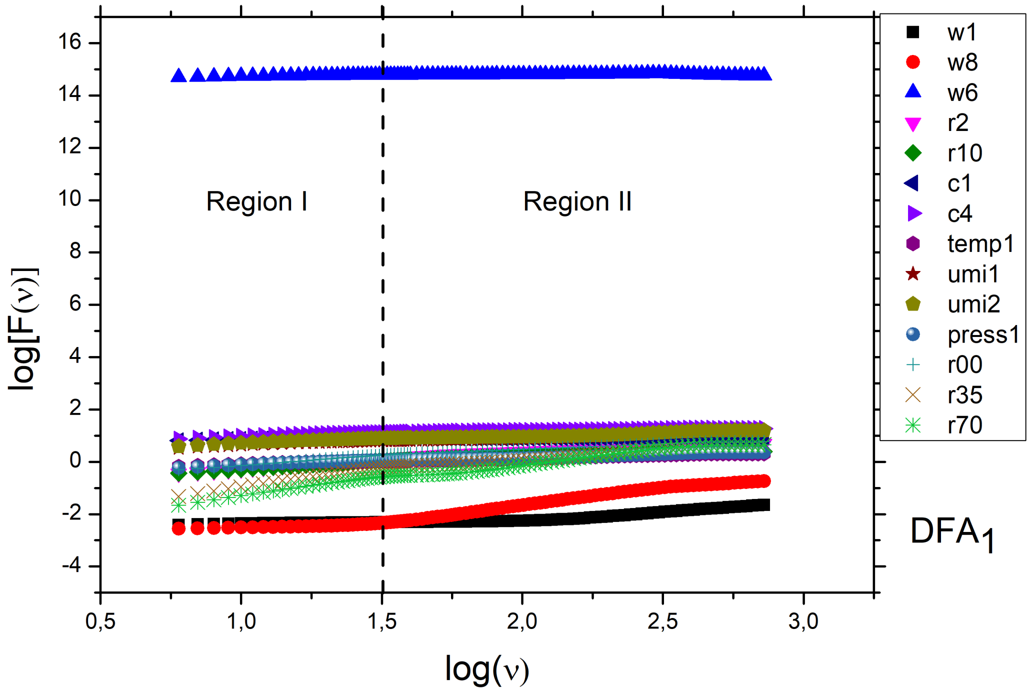

Figure S9. *Detrended Fluctuation analysis around the trend. The colors indicate the attributes. The colors indicate the attributes. Each RI indicates stations summed and weighted by distances to the W1.*

RIMAS code	utme	utmn	Alias
2900020672	464249	8655113	W01
2900020680	367476	8654685	W02
2900020683	419033	8592202	W03
2900020688	466814	8580900	W04
2900021798	397771	8609184	W05
2900024870	430205	8648417	W06
2900024876	508651	8582610	W07
2900024878	428302	8552721	W08

Table S1. *RIMAS code, UTM location and alias used for wells.*

RHN code	utm_e	utm_n	Alias
45760000	449846.2	8481290	RI01
46450500	476245.1	8621740	RI02
46453500	483393.1	8626047	RI03
46455000	486717.9	8628039	RI04
46520000	446729.6	8651902	RI05
46530000	440256	8641627	RI06
46550000	498977.3	8656583	RI07
46570000	433776.2	8684951	RI08
46590000	486930	8689356	RI09
46784000	434421.9	8698654	RI10
46790000	478493.6	8778330	RI11

Table S2. *RHN code, UTM location and alias used for river stations.*

RHN code	utm_e	utm_n	Alias
1145014	486690.2	8689754	RA01
1145024	393834.2	8704899	RA02
1146006	365118.9	8723932	RA03
1245015	397542.7	8588632	RA04
1345002	431598.1	8561088	RA05
1446008	376363	8441223	RA06

Table S3. *RHN code, UTM location and alias used for rain stations.*

INMET code	utm_e	utm_n	Alias
A402	497068.3	8659655	T01/P01/H01
A404	409722.9	8656457	T02/P02/H02
A416	541423.2	8526062	T03/P03/H03
A452	478071.1	8778245	T02/P02/H03

Table S4. *INMET code, UTM location and alias used for sensors in weather stations (temperature, atmospheric pressure, and humidity).*

Method/Time-series	Region I	Region II
DFA_1/Well 1 (W01)	0.115 ± 0.005	0.54 ± 0.02
DFA_1/Well 6 (W06)	0.101 ± 0.005	0.002 ± 0.007
DFA_0/Well 1 (W01)	0.50 ± 0.02	0.976 ± 0.003
DFA_0/Well 6 (W06)	0.049 ± 0.002	0.007 ± 0.007
$DCCA_1$	0.10 ± 0.02	0.132 ± 0.006
$DCCA_0$	0.32 ± 0.01	0.460 ± 0.004

Table S5. *Cross-Correlation exponents considering the Well W01 and Well W06.*

Method/Time-series	Region I	Region II
DFA_1/ River 2 (RI2)	0.781 ± 0.001	0.417 ± 0.005
DFA_1/ River 10 (RI10)	0.638 ± 0.005	0.248 ± 0.005
DFA_0/ River 2 (RI2)	0.628 ± 0.009	0.259 ± 0.007
DFA_0/ River 10 (RI10)	0.457 ± 0.008	0.159 ± 0.003
$DCCA_1$	0.857 ± 0.003	0.434 ± 0.005
$DCCA_0$	0.653 ± 0.005	0.285 ± 0.005

Table S6. *Cross-Correlation exponents considering the River RI2 and River RI10.*

Method/Time-series	Region I	Region II
DFA_1/ Rain 1 (R01)	0.112 ± 0.005	0.037 ± 0.002
DFA_1/ Rain 1 (R04)	0.31 ± 0.01	0.118 ± 0.003
DFA_0/ Rain 1 (R01)	0.056 ± 0.004	0.030 ± 0.002
DFA_0/ Rain 1 (R04)	0.215 ± 0.007	0.079 ± 0.003
$DCCA_1$	0.33 ± 0.01	0.079 ± 0.001
$DCCA_0$	0.219 ± 0.009	0.041 ± 0.001

Table S7. *Cross-Correlation exponents considering the Rain R1 and Rain R4.*

Method/Time-series	Region I	Region II
DFA_1/ Temperature 1 (T01)	0.273 ± 0.008	0.239 ± 0.004
DFA_1/ Humidity 1 (H01)	0.34 ± 0.01	0.337 ± 0.009
DFA_0/ Temperature 1 (T01)	0.195 ± 0.003	0.174 ± 0.006
DFA_0/ Humidity 1 (H01)	0.239 ± 0.004	0.279 ± 0.007
$DCCA_1$	0.34 ± 0.01	0.253 ± 0.007
$DCCA_0$	0.229 ± 0.004	0.236 ± 0.003

Table S8. *Cross-Correlation exponents considering the Temperature T1 and Humidity H1.*

Method/Time-series	Region I	Region II
DFA_1/ Temperature 1 (T01)	0.273 ± 0.008	0.239 ± 0.004
DFA_1/ Humidity 2 (H02)	0.37 ± 0.01	0.257 ± 0.008
DFA_0/ Temperature 1 (T01)	0.195 ± 0.003	0.174 ± 0.006
DFA_0/ Humidity 2 (H02)	0.223 ± 0.006	0.227 ± 0.005
$DCCA_1$	0.38 ± 0.01	0.231 ± 0.007
$DCCA_0$	0.236 ± 0.006	0.223 ± 0.003

Table S9. *Cross-Correlation exponents considering the Temperature T1 and Humidity H2.*

Method/Time-series	Region I	Region II
DFA_1/ Well 1 (W01)	0.115 ± 0.005	0.54 ± 0.02
DFA_1/ Rain 4 (R04)	0.31 ± 0.01	0.118 ± 0.003
DFA_0/ Well 1 (W01)	0.50 ± 0.02	0.976 ± 0.003
DFA_0/ Rain 4 (R04)	0.215 ± 0.007	0.079 ± 0.003
$DCCA_1$	0.30 ± 0.01	0.30 ± 0.01
$DCCA_0$	0.328 ± 0.004	0.610 ± 0.004

Table S10. *Cross-Correlation exponents considering the Well W01 and Rain R4.*

Method/Time-series	Region I	Region II
DFA_1/ Well 1 (W01)	0.115 ± 0.005	0.54 ± 0.02
DFA_1/ River 10 (RI10)	0.638 ± 0.005	0.248 ± 0.005
DFA_0/ Well 1 (W01)	0.50 ± 0.02	0.976 ± 0.003
DFA_0/ River 10 (RI10)	0.457 ± 0.008	0.159 ± 0.003
$DCCA_1$	0.463 ± 0.007	0.411 ± 0.009
$DCCA_0$	0.487 ± 0.001	0.640 ± 0.002

Table S11. *Cross-Correlation exponents considering the Well W01 and River RI10.*

Method/Time-series	Region I	Region II
DFA_1/ Pressure 1 (P01)	0.36 ± 0.01	0.260 ± 0.005
DFA_1/ Temperature 1 (T01)	0.273 ± 0.008	0.239 ± 0.004
DFA_0/ Pressure 1 (P01)	0.238 ± 0.005	0.210 ± 0.006
DFA_0/ Temperature 1 (T01)	0.195 ± 0.003	0.174 ± 0.006
$DCCA_1$	0.35 ± 0.01	0.285 ± 0.007
$DCCA_0$	0.238 ± 0.005	0.250 ± 0.006

Table S12. *Cross-Correlation exponents considering the Pressure P01 and Temperature T01.*

Method/Time-series	Region I	Region II
DFA_1/ Well 1 (W01) Simple Filter	0.115 ± 0.005	0.54 ± 0.02
DFA_1/ River (RI)	0.787 ± 0.005	0.394 ± 0.004
DFA_0/ Well 1 (W01) Simple Filter	0.50 ± 0.02	0.976 ± 0.003
DFA_0/ River (RI)	0.56 ± 0.01	0.252 ± 0.007
$DCCA_1$	0.520 ± 0.002	0.50 ± 0.01
$DCCA_0$	0.559 ± 0.003	0.718 ± 0.004

Table S13. *Cross-Correlation exponents considering the Well W01 and River RI (Simple Filter).*
